# Covariate-assisted bounds on causal effects with instrumental variables

**DOI:** 10.1093/jrsssb/qkaf028

**Published:** 2025-05-27

**Authors:** Alexander W Levis, Matteo Bonvini, Zhenghao Zeng, Luke Keele, Edward H Kennedy

**Affiliations:** Department of Statistics & Data Science, Carnegie Mellon University, Pittsburgh, PA 15213, USA; Department of Statistics & Data Science, Carnegie Mellon University, Pittsburgh, PA 15213, USA; Department of Statistics & Data Science, Carnegie Mellon University, Pittsburgh, PA 15213, USA; Department of Surgery, University of Pennsylvania, Philadelphia, PA 19104, USA; Department of Statistics & Data Science, Carnegie Mellon University, Pittsburgh, PA 15213, USA

**Keywords:** causal inference, instrumental variables, nonparametric bounds, nonparametric efficiency, partial identification

## Abstract

When an exposure of interest is confounded by unmeasured factors, an instrumental variable (IV) can be used to identify and estimate certain causal contrasts. Identification of the marginal average treatment effect (ATE) from IVs relies on strong untestable structural assumptions. When one is unwilling to assert such structure, IVs can nonetheless be used to construct bounds on the ATE. Famously, Alexander Balke and Judea Pearl proved tight bounds on the ATE for a binary outcome, in a randomized trial with noncompliance and no covariate information. We demonstrate how these bounds remain useful in observational settings with baseline confounders of the IV, as well as randomized trials with measured baseline covariates. The resulting bounds on the ATE are nonsmooth functionals, and thus standard nonparametric efficiency theory is not immediately applicable. To remedy this, we propose (1) under a novel margin condition, influence function-based estimators of the bounds that can attain parametric convergence rates when the nuisance functions are modelled flexibly, and (2) estimators of smooth approximations of these bounds. We propose extensions to continuous outcomes, explore finite sample properties in simulations, and illustrate the proposed estimators in an observational study targeting the effect of higher education on wages.

## 1 Introduction

A primary goal in many scientific endeavours is to determine whether an intervention has a causal effect on an outcome. However, simple comparisons between the outcomes of treated and control groups are often complicated by confounding: differences in outcomes between those who are and are not treated due to pretreatment differences rather than the effect of the treatment itself. One solution to selection bias of this form is to allocate the treatment via randomization, rendering pretreatment distributions equal, on average. In many circumstances, however, randomization is infeasible or unethical. When this is the case, one alternative is to identify an instrumental variable (IV). For a variable to be an IV, it must meet the three following conditions: (a) the IV must be associated with the exposure; (b) the IV must be randomly or as-if randomly assigned; and (c) the IV cannot have a direct effect on the outcome (Angrist et al., 1996). Under these conditions, as well as some further structural assumptions, an IV can provide a consistent estimate of a causal effect even in the presence of unobserved confounding between the exposure and the outcome. See Baiocchi et al. (2014) and G. Imbens (2014) for general reviews of IV methods.

In most analyses, investigators focus on point identification—asserting sufficient structure so that a single parameter describing a causal effect can be expressed as a function of the observed data distribution. In fact, assumptions (a)–(c), on their own, are insufficient to point identify an average causal effect. Point identification requires investigators to assume either some form of homogeneity (e.g. lack of effect modification by measured and/or unmeasured confounders) or an assumption known as monotonicity (Angrist et al., 1996; Hernán & Robins, 2006; J. M. Robins, 1994; Tan, 2010). Critically, these structural IV assumptions are (typically) untestable and may be controversial in many applications. One alternative approach is to relax key IV assumptions using partial identification. Under the partial identification approach, analysts seek to estimate bounds on the parameter of interest, which can typically be done with weaker assumptions (Manski, 1990, 1995). There is a large body of work on partial identification in IV designs with foundational work done by, e.g. A. Balke and Pearl (1997) and Manski and Pepper (2000, 2009). See Swanson et al. (2018) for a general overview of partial identification approaches to an IV analysis.

There exists a substantial literature studying bounds on causal effects, incorporating covariate adjustment under various sets of assumptions. Broadly, the use of covariates is beneficial in either (a) experimental settings, where baseline covariate adjustment can improve efficiency (Tsiatis et al., 2008; Yang & Tsiatis, 2001) and bound width (Cai et al., 2007), or (b) observational settings, where confounder adjustment is necessary for key assumptions to hold (Feller et al., 2017; Grilli & Mealli, 2008; Swanson et al., 2018). Notably, Cai et al. (2007) illustrated possible improvements on natural bounds (Manski, 1990) and tight bounds (A. Balke & Pearl, 1997) on average treatment effects (ATEs) in a randomized trial via adjustment of categorical covariates. Long and Hudgens (2013) showed covariate-assisted bounds on principal effects are narrower than the unadjusted bounds. Such adjusted bounds are widely used in principal stratification analysis on evaluating the effects of training and school programs (Lee, 2009; Miratrix et al., 2018). However, most existing work considers the discrete covariate setting (hence plug-in style empirical average estimators are applicable) and does not explore robustness or efficiency theory on the bounds. Richardson et al. (2011) developed a Bayesian approach to estimate bounds in IV settings under a variety of assumptions [including those under which the bounds in A. Balke and Pearl (1997) are derived]. However, these methods rely on strong parametric assumptions on likelihood components (including the covariate density). Similarly, Grilli and Mealli (2008) examined adjusted principal stratification-based bounds using continuous covariates under strong parametric assumptions that can be hard to justify in practice.

In this paper, aiming to address the methodological gaps identified above, we propose and analyse estimators of the Balke–Pearl bounds that can incorporate arbitrarily complex baseline covariate information and avoid strong parametric assumptions. We also provide theory-backed criteria for choosing covariates to adjust for in our approach, so as to reduce bound length. Our proposed estimators are based on semiparametric efficiency theory and use influence functions, which allow for flexible and efficient estimation via a variety of nonparametric machine learning-based methods. The use of influence functions also allows us to derive simple closed-form variance estimators and asymptotically valid confidence intervals. Importantly, our work provides useful insights into estimation strategies for functionals that involve nonsmooth functions of the observed data distribution, and thus are not pathwise differentiable without further assumptions. Such functionals commonly appear when targeting bounds on causal effects (Chesher, 2010; Grilli & Mealli, 2008; Long & Hudgens, 2013; Sachs et al., 2022), for example. In online supplementary material, Appendix S1, we frame the methods proposed in this work more generally for a large class of nonsmooth functionals.

The remainder of the paper is organized as follows. In Section 2, we define notation, review key IV assumptions, and outline our target causal estimand: the ATE. In Section 3, we review extant methods for bounding the ATE based on an IV. We also provide a simple illustration to demonstrate the benefits of incorporating covariate information, and present a general result to guide which covariates to include in the ensuing analyses. Importantly, the nonparametric bounds outlined in Section 3 are nonsmooth functionals of the observed data distribution, so standard semiparametric efficiency theory does not immediately apply. In view of this challenging setting, Sections 4–6 detail the three main methodological contributions of the paper: (i) efficient estimators of the true nonsmooth bound functionals under a margin condition (in Section 4); (ii) valid but conservative bounds on the ATE based on estimators of smooth functional approximations to the bounds (in Section 5); and (iii) extensions of the proposed methods to the case of a general bounded outcome, discrete, or continuous (in Section 6). In Section 7, we present a simulation study to investigate the finite sample properties of the proposed estimators. Finally, in Section 8, we apply our proposed methods to data from an observational study to assess the effects of college education on wages. Proofs of all results can be found in the online supplementary material file.

## 2 Background

### 2.1 Notation and assumptions

Consider the standard IV setup, in which the observed data are *n* iid copies of O=(X,Z,A,Y)∼P, where $X\in X\subseteq R^{d}$ is a vector of covariates, and Z,A∈{0,1} are binary instrument and exposure variables, respectively. Our focus is mostly on binary outcomes, Y∈{0,1}; we will discuss extensions for nonbinary outcomes in Section 6. We let A(z) and Y(z) denote the counterfactual exposure and outcome values, had the instrument been set to Z=z, for z∈{0,1}. Similarly, we also define Y(a) and Y(z,a) to be the potential outcomes under an intervention that sets A=a, and an intervention that sets both Z=z and A=a, respectively. For z∈{0,1}, let $\lambda_{z}(X)=P[Z=z|X]$. Next, we review the set of assumptions that we take as given in the analysis. First, we make the two following assumptions:

Assumption 1 **Consistency**

A(Z)=A , and Y(Z)=Y(A)=Y(Z,A)=Y, almost surely.

Assumption 2 **Positivity**

For some ϵ>0, $\lambda_{1}(X)\in [\epsilon ,1-\epsilon ]$, almost surely.

In words, Assumption 1 asserts that interventions on *Z* and *A* are well-defined, and that there is no interference between subjects, so that a unit’s potential treatment and outcome can be linked to their observed variables; Assumption 2 asserts that either instrument value can be realized, over all strata determined by X. These two assumptions are not unique to the IV framework, and are commonly invoked in many causal inference settings. Next, we make the following ‘core’ IV assumptions:

Assumption 3 **Unconfoundedness**

Z⊥⊥(A(z),Y(z))∣X , for z∈{0,1}.

Assumption 4 **Exclusion Restriction**

Y(z,a)≡Y(a) , for z,a∈{0,1}.

Assumptions 3 and 4, respectively, formalize IV conditions (b) and (c) noted in Section 1, of random assignment and no direct effect of the IV. Namely, Assumption 3 asserts that the effect of *Z* on *A* and *Y* is unconfounded, given measured covariates X. Note that in certain special cases, Assumption 3 holds unconditionally, and we can identify the IV effect without conditioning on X. For example, when *Z* is marginally randomized (e.g. in a trial), Assumption 3 holds by design. Alternatively, in certain natural experiments, analysts may assert that Assumption 3 holds unconditionally, e.g. Angrist and Evans (1998). Assumption 4 asserts that the effect of *Z* on *Y* acts entirely through its effect on *A*, i.e. *Z* has no direct effect on *Y*. Note that most IV studies adopt an assumption of nonzero association between *Z* and *A* [IV condition (a) in Section 1, often referred to as a ‘relevance’ assumption]. However, this assumption is not formally required for partial identification of the ATE.

### 2.2 Estimands and additional structural assumptions

In what follows, we focus on the ATE as the target causal estimand:


E(Y(a=1)−Y(a=0)).

Critically, under the four assumptions introduced in the previous section, the ATE is not point identified. Analysts typically take one of two approaches for point identification. The first approach invokes some type of homogeneity assumptions and places various restrictions on how the effects of *A* and *Z* vary from unit to unit in the study population. See Hernan and Robins (2019) and L. Wang and Tchetgen Tchetgen (2018) for prominent examples. However, homogeneity assumptions are often implausible or difficult to verify in specific applications. The second approach invokes an assumption known as monotonicity, which has the following form: A(z=1)≥A(z=0), i.e. if A(z=0)=1, then A(z=1)=1 (G. W. Imbens & Angrist, 1994). Under monotonicity, the target estimand is typically no longer the ATE, but instead is often the local ATE: E(Y(a=1)−Y(a=0)∣A(z=1)>A(z=0)). Here, investigators must be content with a more local estimand that may not generalize to the ATE. In this paper, we only assume that Assumptions 1–4 hold, and aim to construct tight bounds on the ATE parameter.

## 3 Partial identification for IVs and the role of covariate information

Next, we provide an overview of one partial identification approach for the IV framework. We then provide a brief illustration to demonstrate how covariate information can alter the bounds on the ATE. We also present theoretical results to elucidate when we may expect the inclusion of covariates to tighten the bounds on the ATE.

### 3.1 Review: Balke–Pearl bounds

In their seminal work, Alexander Balke and Judea Pearl leveraged symbolic linear programming to develop sharp nonparametric bounds on the ATE for a binary outcome (A. Balke & Pearl, 1994, 1997; A. A. Balke, 1995). Notably, their bounds only invoke Assumptions 1–4, and are provably tight under these assumptions. In both applications and simulations, they demonstrated substantial narrowing of the bounds on the ATE compared to partial identification results in J. Robins (1989) and Manski (1990).

The results in A. Balke and Pearl (1997) focused exclusively on marginally randomized instruments such that X=∅. As such, their methods do not make use of any measured covariates. Nonetheless, their results hold just as well for bounding the conditional average treatment effect (CATE), E[Y(a=1)−Y(a=0)∣X=x], for any x∈X, in two important settings where Assumptions 1–4 may hold: (i) an experiment with *Z* marginally randomized and baseline covariates X are measured, and (ii) an observational setting where X are baseline confounders required for *Z* to be a valid IV. In case (i), we will demonstrate that incorporating baseline covariate information X can both provide tighter theoretical bounds, and improve statistical precision. In case (ii), conditioning on X is required for the IV assumptions to hold, and thus for the bounds to be valid.

To begin, we review the main theoretical result from A. Balke and Pearl (1997). For each y,a,z∈{0,1}, define $\pi_{ya.z}(X)=P(Y=y,A=a|X,Z=z)$. Moreover, define the two 8-dimensional vectors $\theta_{\ell }(X)=(\theta_{\ell ,1}(X),\ldots ,\theta_{\ell ,8}(X)),\theta_{u}(X)=(\theta_{u,1}(X),\ldots ,\theta_{u,8}(X))\in [-1,1{]}^{8}$, where omitting inputs,


(1) $$\begin{array}{rl} \theta_{\ell ,1} & =\pi_{11.1}+\pi_{00.0}-1,\\ \theta_{\ell ,2} & =\pi_{11.0}+\pi_{00.1}-1,\\ \theta_{\ell ,3} & =-\pi_{01.1}-\pi_{10.1},\\ \theta_{\ell ,4} & =-\pi_{01.0}-\pi_{10.0},\\ \theta_{\ell ,5} & =\pi_{11.0}-\pi_{11.1}-\pi_{10.1}-\pi_{01.0}-\pi_{10.0},\\ \theta_{\ell ,6} & =\pi_{11.1}-\pi_{11.0}-\pi_{10.0}-\pi_{01.1}-\pi_{10.1},\\ \theta_{\ell ,7} & =\pi_{00.1}-\pi_{01.1}-\pi_{10.1}-\pi_{01.0}-\pi_{00.0},\\ \theta_{\ell ,8} & =\pi_{00.0}-\pi_{01.0}-\pi_{10.0}-\pi_{01.1}-\pi_{00.1} \end{array}$$

and


(2) $$\begin{array}{rl} \theta_{u,1} & =1-\pi_{01.1}-\pi_{10.0},\\ \theta_{u,2} & =1-\pi_{01.0}-\pi_{10.1},\\ \theta_{u,3} & =\pi_{11.1}+\pi_{00.1},\\ \theta_{u,4} & =\pi_{11.0}+\pi_{00.0},\\ \theta_{u,5} & =-\pi_{01.0}+\pi_{01.1}+\pi_{00.1}+\pi_{11.0}+\pi_{00.0},\\ \theta_{u,6} & =-\pi_{01.1}+\pi_{11.1}+\pi_{00.1}+\pi_{01.0}+\pi_{00.0},\\ \theta_{u,7} & =-\pi_{10.1}+\pi_{11.1}+\pi_{00.1}+\pi_{11.0}+\pi_{10.0},\\ \theta_{u,8} & =-\pi_{10.0}+\pi_{11.0}+\pi_{00.0}+\pi_{11.1}+\pi_{10.1}. \end{array}$$

Finally, define $\gamma_{\ell }(X)=\max_{1\leq j\leq 8}\theta_{\ell ,j}(X)$ and $\gamma_{u}(X)=\min_{1\leq j\leq 8}\theta_{u,j}(X)$. Balke and Pearl proved [e.g. see the main result in A. Balke and Pearl (1997), p. 1173] that the CATE is bounded between $\gamma_{\ell }$ and $\gamma_{u}$, the tightest possible lower and upper bounds, respectively, under Assumptions 1–4. We summarize their result in the following theorem.

Theorem 1 **A. Balke and Pearl (1997)**

Under Assumptions 1–4, for $(Z,A,Y)\in \{0,1{\}}^{3}$, the CATE can be bounded as

$$\gamma_{\ell }(X)\leq E(Y(a=1)-Y(a=0)|X)\leq \gamma_{u}(X).$$

These bounds are tight in the nonparametric model. Moreover, marginalizing yields bounds on the ATE:

(3) $$E_{P}(\gamma_{\ell }(X))\leq E(Y(a=1)-Y(a=0))\leq E_{P}(\gamma_{u}(X)),$$

which are also tight in the nonparametric model.

The primary goal of this paper is to construct efficient estimators of the tight bounds on the ATE given in Theorem 1, $L(P)\;:\;=E_{P}(\gamma_{\ell }(X))$ and $U(P)\;:\;=E_{P}(\gamma_{u}(X))$. Statistically, this is a challenging task, since these are means of a pointwise maximum and minimum, each of which is a nonsmooth function. We will outline two strategies to deal with this difficulty: (1) invoking a margin condition (see Section 4), or (2) targeting a smooth approximation to the Balke–Pearl bounds (see Section 5).

Before describing strategies for estimation, we first demonstrate the benefit of pursuing covariate-assisted bounds. In Section 3.2, we give a simple illustration in the randomized experimental setting, showing that covariate-assisted bounds can be substantially narrower than unadjusted bounds. In Section 3.3, we provide a general result to guide the choice of adjustment set X when more than one is possible.

### 3.2 Motivating illustration

Consider a hypothetical randomized experiment with arm assignment Z∼Bernoulli(0.5), independent of baseline covariates $X_{1}∼\mathrm{Bernoulli}(0.7)$, $X_{2}∼\mathrm{Unif}(-1,1)$, with $X_{1}⊥⊥X_{2}$. In this example, $X_{1}$ represents a behavioural or demographic factor, and $X_{2}$ represents an underlying risk score (i.e. low values represent good health and low risk, high values represent poor health and high risk). In this experiment, we suppose that there is a degree of noncompliance, which is completely determined by the covariates $X_{1}$ and $X_{2}$. Specifically, we set:


$$\begin{array}{rl} A(z=0)A(z=1) & =1(X_{2}\geq 0.99),\\ \left\{1-A(z=0)\}\{1-A(z=1)\right\} & =1(X_{2}\leq -0.99),\\ A(z=0)\{1-A(z=1)\} & =(1-X_{1})1(X_{2}\in (-0.5,0.5]),\\ \left\{1-A(z=0)\}A(z=1\right) & =1-1(|X_{2}|\geq 0.99)-(1-X_{1})1(X_{2}\in (-0.5,0.5]). \end{array}$$

In words, we can define four principal strata: ‘always takers’, who are exposed regardless of instrument status, are those with the very highest underlying risk; ‘never takers’, who are not exposed regardless of instrument status, are those with the very lowest risk; ‘defiers’, whose exposure value is opposite to that of the instrument, are those with intermediate risk and behavioural factor $X_{1}=0$; and ‘compliers’, whose exposure value matches that of the instrument, comprise the remainder of the population (Angrist et al., 1996; Frangakis & Rubin, 2002). We further suppose the potential outcomes Y(a) are completely determined by the compliance classes U=(A(z=0),A(z=1)), and set $Y(a)|U∼\mathrm{Bernoulli}(p_{a}(U))$, with


$$p_{a}=\left\{ \begin{array}{cc} 0.20+0.10a, & \text{if }A(z=0)A(z=1)=1,\\ 0.90+0.05a, & \text{if }\{1-A(z=0)\}\{1-A(z=1)\}=1,\\ 0.65+0.05a, & \text{if }A(z=0)\{1-A(z=1)\}=1,\\ 0.25+0.10a, & \text{if }\{1-A(z=0)\}A(z=1)=1. \end{array}\right..$$

**Figure 1.**
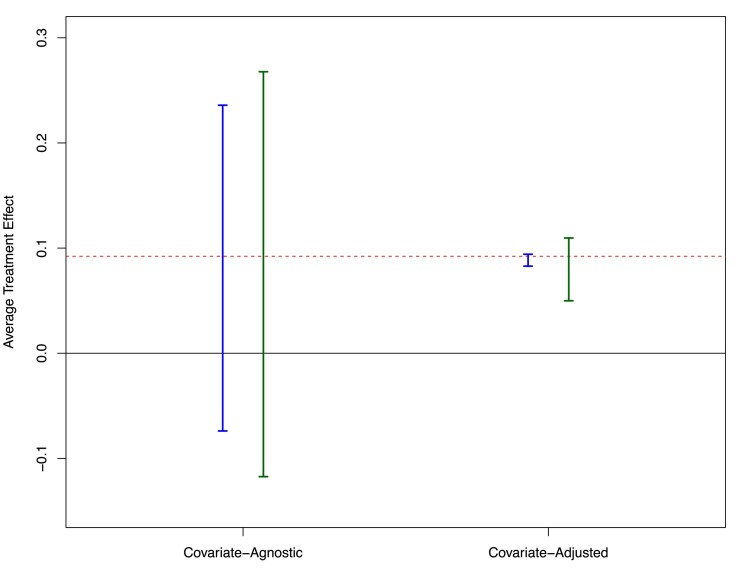
Balke–Pearl bounds for the average treatment effect (ATE), with and without covariate adjustment. The dashed horizontal line represents the true ATE. Left (blue) bars represent the true theoretical bounds, and right (green) bars are estimated 95% confidence intervals from a single sample of 5,000 subjects (with 10-fold cross-fitting for adjusted approach). The solid horizontal line indicates the reference value of zero average effect.

Figure 1 shows the covariate-agnostic and covariate-adjusted Balke–Pearl bounds on the ATE resulting from Theorem 1. The true bounds are plotted in blue and estimated 95% confidence intervals (from one simulated sample of size 5,000) for the bounds are plotted in green. The covariate-agnostic bounds, while simple to compute as the maximum and minimum of eight (true or empirical) probabilities, are quite wide and cover the null treatment effect of zero. The covariate-adjusted bounds, on the other hand, are very narrow and are bounded away from zero. Employing the estimator, we propose in Section 4, with flexible regression tree-based nuisance function estimation, we obtain valid and narrow estimated bounds on the ATE.

Note that in this example, the covariates $X_{1}$, $X_{2}$ happen to comprise all confounders of the relationship between *A* and *Y*, so that direct adjustment (e.g. with the *g*-formula) would identify the ATE. A practitioner without this knowledge, but with the foresight to measure $X_{1}$ and $X_{2}$, could still use these covariates to construct informative bounds. We also note that our example is somewhat extreme, as we would not typically expect the covariate-adjusted bounds to always be so narrow. However, we show below that in this experimental design, the covariate-adjusted bounds are guaranteed to be at least as narrow as unadjusted bounds, even in the presence of residual exposure-outcome confounding given X. Indeed, one will want to adjust for X whenever these covariates are predictive of *A* and *Y*.

### 3.3 Width of the bounds

In the illustration above, both choices of adjustment sets [no covariates or $\left(X_{1},X_{2}\right)$] result in valid bounds since Assumptions 1–4 are satisfied with either choice. In our example, the covariate-assisted Balke–Pearl bounds improve substantially upon naïve covariate-agnostic bounds. In fact, such additional adjustment in general cannot do worse, and can only improve upon unadjusted bounds. We formalize this idea in the following result.

Proposition 1 Suppose X renders *Z* a valid instrument according to Assumptions 1–4. Suppose that Assumptions 2 and 3 also hold after augmenting X with G, and Z⊥⊥G∣X, i.e. G does not predict *Z*, except possibly through association with X. Then, Balke–Pearl bounds based on (X,G) are at least as narrow as those based on X, and may be strictly narrower.

According to Proposition 1, we would want to include in X any pure predictors of (A,Y) that are not direct confounders of the effect of *Z* on *A* and *Y*, when constructing Balke–Pearl bounds. This incentivizes measurement and inclusion of residual exposure-outcome confounders beyond the conventional wisdom in observational studies. For instance, in a randomized trial subject to noncompliance, one would want to measure and include any known predictors of compliance status, e.g. in many clinical applications, health-seeking behaviour. The result of Proposition 1 bears some resemblance to Lemma 4 in Rotnitzky and Smucler (2020), which says that influence function-based estimation of the ATE from an observational study is improved by adding pure outcome predictors—so-called ‘precision variables’—to an already sufficient set of confounders. Note, however, that in that setting improvement corresponded to lower variance, whereas here we are concerned both with the width of the theoretical bounds, as well as the variance of our estimators. Specializing Proposition 1 to the case where *Z* is marginally randomized yields the following corollary:

Corollary 1 Suppose Assumptions 1–4 hold, Z⊥⊥X, and *Z* is also marginally unconfounded, i.e. Z⊥⊥(A(z),Y(z)) for z∈{0,1}. Then, Balke–Pearl bounds based on X are at least as narrow as unadjusted bounds.

Corollary 1 explains what we observed in the motivating example of Section 3.2. That is, a marginally randomized IV guarantees that inclusion of baseline covariates X will result in bounds that are no worse than unadjusted bounds. The degree to which such inclusion narrows the bounds is of great interest, and will help in deciding which covariates to measure in practice—a more precise characterization of this improvement we leave for future research. For the remainder of the paper, we focus on constructing valid and efficient estimators of the covariate-assisted Balke–Pearl bounds.

## 4 Bound estimation under a margin condition

As mentioned in Section 3.1, the bound functionals $L(P)=E_{P}(\gamma_{\ell }(X))$ and $U(P)=E_{P}(\gamma_{u}(X))$ are not pathwise differentiable without further restrictions, and therefore do not have an influence functions to enable estimation as described above. This is because the pointwise maximum, $\gamma_{\ell }(X)=\max_{1\leq j\leq 8}\theta_{\ell ,j}(X)$, and pointwise minimum, $\gamma_{u}(X)=\min_{1\leq j\leq 8}\theta_{u,j}(X)$, are not differentiable everywhere. Indeed, we should not expect it to be possible in the nonparametric model to estimate the covariate-adjusted bounds at parametric rates. In this section, we introduce an additional assumption known as a margin condition that renders the bounds pathwise differentiable. Further, we describe estimators of the bounds that exploit the margin condition in order to achieve faster rates. For a broader overview of the use of influence functions for efficient and robust estimation, see online supplementary material, Appendix S4.

### 4.1 An infeasible estimator

To motivate the proposed estimator, we first consider an infeasible estimator of the bounds. Let $d_{\ell }(X)\in \arg\;\;\max_{1\leq j\leq 8}\theta_{\ell ,j}(X)$ and $d_{u}(X)\in \arg\;\;\min_{1\leq j\leq 8}\theta_{u,j}(X)$. The bounds can thus be written as


$$L(P)=\sum_{\;j=1}^{8}E\left[1\{d_{\ell }(X)=j\}\theta_{\ell ,j}(X)\right]\;\text{and}\;U(P)=\sum_{\;j=1}^{8}E\left[1\{d_{u}(X)=j\}\theta_{u,j}(X)\right].$$

For each y,a,z∈{0,1}, define


$$\psi_{ya.z}(O;P)=\frac{1(Z=z)}{\lambda_{z}(X)}\left\{1(Y=y,A=a)-\pi_{ya.z}(X)\right\}.$$

Under the assumption that $x\mapsto d_{\ell }(x)$ and $x\mapsto d_{u}(x)$ are known functions, and are unique maximizers and minimizers, respectively, it can be shown that the uncentred influence functions of L(P) and U(P) are


$$\begin{array}{rl} & \varphi_{\ell }(O;P,d_{\ell })=\sum_{\;j=1}^{8}1\{d_{\ell }(X)=j\}\left\{L_{j}(O;P)+\theta_{\ell ,j}(X)\right\},\\ & \varphi_{u}(O;P,d_{u})=\sum_{\;j=1}^{8}1\{d_{u}(X)=j\}\left\{U_{j}(O;P)+\theta_{u,j}(X)\right\}, \end{array}$$

where $L_{j}(O;P)$ and $U_{j}(O;P)$ are obtained by replacing $\pi_{ya.z}(X)$ with $\psi_{ya.z}(O;P)$ in $\theta_{\ell ,j}(X)$ and $\theta_{u,j}(X)$ in display (1), respectively, and omitting the constant 1 whenever it appears, e.g.


$$L_{1}=\psi_{11.1}+\psi_{00.0},\;U_{1}=-\psi_{01.1}-\psi_{10.0}.$$

Therefore, under minor regularity conditions, an ‘infeasible’ estimator $\tilde{L}=P_{n}\{\varphi_{\ell }(O;\hat{P},d_{\ell })\}$, i.e. requiring complete knowledge of $d_{\ell }$, would satisfy


$$\sqrt{n}(\tilde{L}-L(P))\;⇝\;N(0,{\mathrm{Var}}_{P}\{\varphi_{\ell }(O;P,d_{\ell })\})$$

as long as $P\{\varphi_{\ell }(O;P,d_{\ell })-\varphi_{\ell }(O;\hat{P},d_{\ell })\}=o_{P}(n^{-1/2})$. In this regime, $\tilde{L}$ would be efficient.

### 4.2 Estimation and inference

In light of the discussion in the section above, we propose estimating the bounds with


$$\begin{array}{rl} & \hat{L}=\sum_{\;j=1}^{8}P_{n}\left[1\{{\hat{d}}_{\ell }(X)=j\}\{L_{j}(O;\hat{P})+{\hat{\theta }}_{\ell ,j}(X)\}\right]=P_{n}\{\varphi_{\ell }(O;\hat{P},{\hat{d}}_{\ell })\},\\ & \hat{U}=\sum_{\;j=1}^{8}P_{n}\left[1\{{\hat{d}}_{u}(X)=j\}\{U_{j}(O;\hat{P})+{\hat{\theta }}_{u,j}(X)\}\right]=P_{n}\{\varphi_{u}(O;\hat{P},{\hat{d}}_{u})\}. \end{array}$$

That is, to estimate the nonsmooth components of the bounds L(P) and U(P), namely the indicators $1\{d_{\ell }(X)=j\}$ and $1\{d_{u}(X)=j\}$, we use plug-in estimators $1\{{\hat{d}}_{\ell }(X)=j\}$ and $1\{{\hat{d}}_{u}(X)=j\}$, where ${\hat{d}}_{\ell }\in \arg\;\;\max_{1\leq j\leq 8}{\hat{\theta }}_{\ell ,j}$ and ${\hat{d}}_{u}\in \arg\;\;\min_{1\leq j\leq 8}{\hat{\theta }}_{u,j}$. A natural question is then under what conditions, if any, the estimators $\hat{L}$ and $\hat{U}$ behave, at least asymptotically, like their infeasible counterparts $\tilde{L}$ and $\tilde{U}$. As shown in the next theorem, a sufficient condition for such oracle behaviour is captured by the ‘margin’ condition below. The pointwise minimum (maximum) is nondifferentiable at points where multiple components attain the minimum (maximum), e.g. $\min\left(x_{1},x_{2}\right)$ is nondifferentiable on the set $\left\{(x_{1},x_{2})\in R\;:\;x_{1}=x_{2}\right\}$. Thus, intuitively, the estimands are not pathwise differentiable when there is significant mass around these ‘ties’. The following additional assumption controls the probability that the minimum and maximum are near these points of nondifferentiability.

Assumption 5 **Margin condition**

There exists α>0 such that for any t≥0,

(4) $$P\left[\min_{\;j\neq d_{\ell }(X)}\{\theta_{\ell ,d_{\ell }(X)}(X)-\theta_{\ell ,j}(X)\}\leq t\right]≲t^{\alpha }$$

and

(5) $$P\left[\min_{\;j\neq d_{u}(X)}\{\theta_{u,j}(X)-\theta_{u,d_{u}(X)}(X)\}\leq t\right]≲t^{\alpha }.$$

The margin condition in Assumption 5 is very similar to conditions that have been proposed and leveraged in the classification literature (Audibert & Tsybakov, 2007), as well as in dynamic treatment regimes (Luedtke & van der Laan, 2016; Qian & Murphy, 2011) and other IV problems (Kennedy et al., 2020). In words, condition (4) says that with high probability, $\theta_{\ell ,d_{\ell }}$ is separated from nonmaximal lower bound values $\theta_{\ell ,j}$. Similarly, condition (5) limits how close nonminimal upper bound values $\theta_{u,j}$ are to the actual minimum $\theta_{u,d_{u}}$. If $\min_{\;j\neq d_{\ell }(X)}\{\theta_{\ell ,d_{\ell }(X)}(X)-\theta_{\ell ,j}(X)\}$, for instance, has bounded density near zero, then (4) will hold with α=1. This is a relatively weak requirement, which we expect to hold in many cases. Note that, setting t=0 in Assumption 5, we are assuming that the maximizer $d_{\ell }$ and minimizer $d_{u}$ are almost surely unique, which avoids some issues that would arise when ties happen with positive probability (Luedtke & van der Laan, 2016). The plausibility of this restriction must be verified on a case-by-case basis, but in general we think the occurrence of ties to be unlikely in real settings. Under Assumption 5, we are able to derive sufficient conditions such that $\hat{L}$ and $\hat{U}$ are $\sqrt{n}$-consistent and asymptotically normal.

Theorem 2 Suppose that the nuisance functions ${\hat{\pi }}_{ya.z}$ and ${\hat{\lambda }}_{z}$ are estimated from a separate independent sample. Moreover, suppose that Assumption 5 holds, $P(\epsilon \leq {\hat{\lambda }}_{1}(X)\leq 1-\epsilon )=1$, for some ϵ>0, $\left\|{\hat{\lambda }}_{1}-\lambda_{1}\|=o_{P}(1\right)$, and $\max_{y,a,z\in \{0,1\}}\|{\hat{\pi }}_{ya.z}-\pi_{ya.z}\|=o_{P}(1)$. Then, we have

$$\begin{array}{rl} \hat{L}-L & =(P_{n}-P)\varphi_{\ell }(O;P,d_{\ell })\\ & \;+O_{P}\left(\left\|{\hat{\lambda }}_{1}-\lambda_{1}\right\|\cdot \max_{y,a,z\in \{0,1\}}\left\|{\hat{\pi }}_{ya.z}-\pi_{ya.z}\right\|+\max_{1\leq j\leq 8}{\left\|{\hat{\theta }}_{\ell ,j}-\theta_{\ell ,j}\right\|}_{\infty }^{1+\alpha }\right)\\ & \;+o_{P}(n^{-1/2}) \end{array}$$

and

$$\begin{array}{rl} \hat{U}-U & =(P_{n}-P)\varphi_{u}(O;P,d_{u})\\ & \;+O_{P}\left(\left\|{\hat{\lambda }}_{1}-\lambda_{1}\right\|\cdot \max_{y,a,z\in \{0,1\}}\left\|{\hat{\pi }}_{ya.z}-\pi_{ya.z}\right\|+\max_{1\leq j\leq 8}{\left\|{\hat{\theta }}_{u,j}-\theta_{u,j}\right\|}_{\infty }^{1+\alpha }\right)\\ & \;+o_{P}(n^{-1/2}). \end{array}$$

In this result, we assume the nuisance functions are estimated on separate independent data for simplicity: in practice with one random sample, one can split the sample and use cross-fitting to achieve the same asymptotic behaviour (Chernozhukov et al., 2018; Zheng & van der Laan, 2010). Sample splitting allows us to avoid complicated empirical process conditions (Chernozhukov et al., 2018; Kennedy et al., 2020) and derive bounds on the conditional bias of the estimator in terms of the convergence rate of the nuisance functions.

Note that in Theorem 2, we do not require the individual nuisance functions to converge at $\sqrt{n}$-rates. The conditions are on the product of convergence rates, or rates raised to a power >1. This is a key advantage of a robust estimator: after we correct for the first-order bias, the remaining bias only involves higher-order terms and is much smaller. The result of Theorem 2 establishes that $\hat{L}$, for instance, is $\sqrt{n}$-consistent as long as


$$\left\|{\hat{\lambda }}_{1}-\lambda_{1}\right\|\cdot \max_{y,a,z\in \{0,1\}}\left\|{\hat{\pi }}_{ya.z}-\pi_{ya.z}\right\|+\max_{1\leq j\leq 8}{\left\|{\hat{\theta }}_{\ell ,j}-\theta_{\ell ,j}\right\|}_{\infty }^{1+\alpha }=o_{P}(n^{-1/2}).$$

The first product-bias term results from estimation of $\theta_{\ell ,j}$ with $L_{j}(O;\hat{P})+{\hat{\theta }}_{\ell ,j}$. The second term denotes a bound on the bias arising plug-in estimation of the indicators $1\{d_{\ell }(x)=j\}$ and depends on the exponent *α* from Assumption 5. For example, as long as the quantity $\min_{\;j\neq d_{\ell }(X)}\{\theta_{\ell ,d_{\ell }(X)}-\theta_{\ell ,j}(X)\}$ has a bounded density near zero, then Assumption 5 holds with α=1. In this case, $\max_{y,a,z\in \{0,1\}}\|{\hat{\pi }}_{ya.z}-\pi_{ya.z}{\|}_{\infty }=o_{P}(n^{-1/4})$ is typically sufficient to imply


$$\max_{1\leq j\leq 8}{\left\|{\hat{\theta }}_{\ell ,j}-\theta_{\ell ,j}\right\|}_{\infty }^{1+\alpha }=o_{P}(n^{-1/2}).$$

Finally, Theorem 2 outlines sufficient conditions for the asymptotic normality of $\hat{L}$ and $\hat{U}$ so that Wald-type confidence intervals are straightforward to compute. In particular, under the conditions for $\sqrt{n}$-consistency, we can construct an asymptotically valid 100(1−δ)% confidence interval for the ATE with


$$\left(\hat{L}-z_{1-\delta /2}\sqrt{\hat{V}/n},\;\hat{U}+z_{1-\delta /2}\sqrt{\hat{W}/n}\right),$$

where $\hat{V}=P_{n}[\{\varphi_{\ell }(O;\hat{P},{\hat{d}}_{\ell })-\hat{L}{\}}^{2}]$, $\hat{V}=P_{n}[\{\varphi_{u}(O;\hat{P},{\hat{d}}_{u})-\hat{U}{\}}^{2}]$, and where $z_{\beta }$ is the *β*th quantile of the standard normal distribution. Moreover, the procedure proposed in G. W. Imbens and Manski (2004) can be used to conduct more precise inferences (see also Theorem 3 in Jiang & Ding, 2018).

We highlight that marginally randomized instruments present a special case of interest. In this case, Z⊥⊥X holds by randomization of treatment assignment *Z* and $\lambda_{z}(X)$ is equal to a known constant $\lambda_{z}$ by design. Here, the requirement for the convergence rate is reduced to $\max_{1\leq j\leq 8}\|{\hat{\theta }}_{\ell ,j}-\theta_{\ell ,j}{\|}_{\infty }=o_{P}(n^{-\frac{1}{2(1+\alpha )}})$.

## 5 Targeting smooth approximations

As an alternative to direct estimation of the bounds under a margin condition, our second proposal is to instead target approximations to the bounds, $L_{g}(P)=E_{P}(g(\theta_{\ell }(X)))$ and $U_{h}(P)=E_{P}(h(\theta_{u}(X)))$, where $g,h\;:\;[-1,1{]}^{8}\to R$ are sufficiently smooth approximate pointwise maximum and minimum functions, respectively.

### 5.1 Approximation based on the log-sum-exp function

While other approximations are possible, we will focus on the log-sum-exp (LSE) function as an approximate maximum. Namely, for any t>0, define $g_{t}\;:\;R^{k}\to R$ via


(6) $$g_{t}(v)=\frac{1}{t}\log\;\left(\sum_{\;j=1}^{k}e^{tv_{j}}\right),\;\;\text{for }v\in R^{k}.$$

The LSE is a smooth convex function (Boyd et al., 2004), with gradient and Hessian given by


$$\nabla g_{t}(v)=\frac{z}{1^{T}z},\;\nabla^{2}g_{t}(v)=\frac{t}{{\left(1^{T}z\right)}^{2}}\left[\left(1^{T}z\right)\text{diag}(z)-zz^{T}\right],$$

where $z=(e^{tv_{1}},\ldots ,e^{tv_{k}})$. Importantly, for our purposes, the following inequality shows that LSE approximates the pointwise maximum function:


$$\max\left\{v_{1},\ldots ,v_{k}\right\}<g_{t}(v)\leq \max\left\{v_{1},\ldots ,v_{k}\right\}+\frac{\log\;k}{t}.$$

Increasing the tuning parameter *t* yields smaller approximation error. On the other hand, the Hessian matrix of $g_{t}$ also depends critically on *t*; as we will see in the following discussions, as *t* increases, the operator norm $\|\nabla^{2}g_{t}(v){\|}_{\mathrm{op}}$ increases, which may induce larger estimation error.

In the context of our problem, we replace the maximum function in $E_{P}(\max_{1\leq j\leq 8}\theta_{\ell ,j}(X))$ with the LSE function and focus on estimating the smooth functional $E_{P}(g_{t}(\theta_{\ell }(X)))$. By the approximation property, $E_{P}(g_{t}(\theta_{\ell }(X)))$ satisfies


$$E\left(\max_{1\leq j\leq 8}\theta_{\ell ,j}(X)\right)\leq E(g_{t}(\theta_{\ell }(X)))\leq E\left(\max_{1\leq j\leq 8}\theta_{\ell ,j}(X)\right)+\frac{\log\;8}{t}.$$

We can similarly define a smooth approximation for pointwise minimum function as $h_{t}=g_{-t}$ and estimate the smooth functional $E_{P}(h_{t}(\theta_{u}(X)))$ for the upper bound $E_{P}(\min_{1\leq j\leq 8}\theta_{u,j}(X))$. The smooth approximation for the minimum function satisfies


$$E\left(\min_{1\leq j\leq 8}\theta_{u,j}(X)\right)-\frac{\log\;8}{t}\leq E(h_{t}(\theta_{u}(X)))\leq E\left(\min_{1\leq j\leq 8}\theta_{u,j}(X)\right).$$

In the remaining part of this section, we develop efficiency theory for these smooth functional approximations of the Balke–Pearl bounds, and propose robust and efficient estimators.

### 5.2 Efficiency theory

In the following theorem, we present the nonparametric efficient influence function for functionals of the form $L_{g}(P)=E_{P}(g(\theta_{\ell }(X)))$ and $U_{h}(P)=E_{P}(h(\theta_{u}(X)))$, where $g,h\;:\;[-1,1{]}^{8}\mapsto R$ are smooth functions with continuous first-order derivatives.

Theorem 3 The nonparametric influence function of $L_{g}(P)$ is

$${\dot{L}}_{g}(O;P)=g\left(\theta_{\ell }(X)\right)-L_{g}(P)+\sum_{\;j=1}^{8}\frac{\partial g\left(\theta_{\ell }(X)\right)}{\partial \theta_{\ell ,j}(X)}L_{j}(O;P),$$

where $L_{j}(O;P)$ is defined in Section 4.1. Similarly, the nonparametric influence function of $U_{h}(P)$ is

$${\dot{U}}_{h}(O;P)=h\left(\theta_{u}(X)\right)-U_{h}(P)+\sum_{\;j=1}^{8}\frac{\partial h\left(\theta_{u}(X)\right)}{\partial \theta_{u,j}(X)}U_{j}(O;P),$$

again with $U_{j}(O;P)$ defined in Section 4.1.

Theorem 3 implies that there are two terms contributing to the influence functions (and hence our proposed estimators to follow) of the smooth functionals $L_{g}(P)$ and $U_{h}(P)$. The first term for the lower bound, $g(\theta_{\ell }(X))-L_{g}(P)$, is augmented with the second term, $\sum_{\;j=1}^{8}\frac{\partial g(\theta_{\ell }(X))}{\partial \theta_{\ell ,j}(X)}L_{j}(O;P)$, which effectively reduces bias that results from estimating the unknown functions $\pi_{ya.z}$ and $\lambda_{z}$.

After characterizing the influence functions for $L_{g}(P)$ and $U_{h}(P)$, we can use them to correct for the first-order bias in the von Mises expansion and arrive at robust estimators, which allows us to perform estimation and inference efficiently.

### 5.3 Estimation and inference

Next, we propose and analyse a robust estimator for the smooth lower bound functional, $L_{g}(P)$. Similar results hold for $U_{h}(P)$ using the same arguments. As in Section 4, we assume that we train models for the nuisance functions $\pi_{ya.z}(X),\lambda_{z}(X)$ based on a separate independent sample $D^{n}$. The robust estimator is defined as:


$${\hat{L}}_{g}=L_{g}(\hat{P})+P_{n}\left[{\dot{L}}_{g}(O;\hat{P})\right]=P_{n}\left[g\left({\hat{\theta }}_{\ell }(X)\right)+\sum_{\;j=1}^{8}\frac{\partial g\left({\hat{\theta }}_{\ell }(X)\right)}{\partial {\hat{\theta }}_{\ell ,j}(X)}L_{j}(O;\hat{P})\right].$$

The following theorem characterizes the conditional bias (given the training data) of the robust estimator ${\hat{L}}_{g}$ and establishes its asymptotic normality under additional conditions.

Theorem 4 Suppose $g\;:\;R^{8}\mapsto R$ is twice continuously differentiable, such that $\|\nabla g(\theta ){\|}_{\infty }\leq C_{1}$ and $\|\nabla^{2}g(\theta ){\|}_{\mathrm{op}}\leq C_{2}$ uniformly over θ, and the nuisance functions ${\hat{\pi }}_{ya.z},{\hat{\lambda }}_{z}$ are estimated from a separate independent sample, $D^{n}$. Moreover, suppose there exists positive constant ϵ such that

$$P\left(\epsilon \leq {\hat{\lambda }}_{1}(X)\leq 1-\epsilon \right)=1.$$

Then, the conditional bias of the robust estimator ${\hat{L}}_{g}$ can be bounded as

$$\begin{array}{rl} & \left|E[{\hat{L}}_{g}|D^{n}]-L_{g}(P)\right|\\ & \;≲\left(\max_{y,a,z\in \{0,1\}}\left\|{\hat{\pi }}_{ya.z}-\pi_{ya.z}\right\|\right)\left(C_{1}\left\|{\hat{\lambda }}_{1}-\lambda_{1}\right\|+C_{2}\max_{y,a,z\in \{0,1\}}\left\|{\hat{\pi }}_{ya.z}-\pi_{ya.z}\right\|\right). \end{array}$$

Let $f(O)={\dot{L}}_{g}(O;P)+L_{g}(P)$ be the noncentred influence function. If we further assume $\left\|\hat{\;f}-f\|=o_{P}(1\right)$ and the nuisance estimators satisfy

$$\begin{array}{rl} & \left\|{\hat{\lambda }}_{1}-\lambda_{1}\right\|\left(\max_{y,a,z\in \{0,1\}}\left\|{\hat{\pi }}_{ya.z}-\pi_{ya.z}\right\|\right)=o_{P}(n^{-1/2}),\\ & \max_{y,a,z\in \{0,1\}}{\left\|{\hat{\pi }}_{ya.z}-\pi_{ya.z}\right\|}^{2}=o_{P}(n^{-1/2}), \end{array}$$

then we have

$${\hat{L}}_{g}-L_{g}(P)=P_{n}\left[{\dot{L}}_{g}(O;P)\right]+o_{P}(n^{-1/2}),$$

implying the robust estimator is $\sqrt{n}$-consistent and achieves the nonparametric efficiency bound.

Similar to the analysis following Theorems 2 and 4 implies that if the nuisance estimators satisfy $\left\|{\hat{\lambda }}_{1}-\lambda_{1}\|=O_{P}(n^{-1/4}\right)$ and $\max_{y,a,z\in \{0,1\}}\|{\hat{\pi }}_{ya.z}-\pi_{ya.z}\|=o_{P}(n^{-1/4})$, the robust estimator will be $\sqrt{n}$-consistent and achieve the efficiency bound. Such nuisance error rates are attainable by a variety of flexible regression methods under various forms of underlying structure. If $\lambda_{1}$, for instance, belongs to a *s*-Hölder class, and ${\hat{\lambda }}_{1}$ is an appropriately tuned series- or local polynomial-based estimator, the minimax optimal rate $\left\|{\hat{\lambda }}_{1}-\lambda_{1}\|=O_{P}(n^{-\frac{1}{2+d/s}}\right)$ can be achieved (Györfi et al., 2006; Tsybakov, 2009). In this case, the desired condition $\left\|{\hat{\lambda }}_{1}-\lambda_{1}\|=O_{P}(n^{-1/4}\right)$ will hold if s>d/2. Alternatively, if $\lambda_{1}$ is an *s*-sparse linear model, the minimax rate $\left\|{\hat{\lambda }}_{1}-\lambda_{1}\|=O_{P}(\sqrt{\frac{s\log\;\left(d\right)}{n}}\right)$ can be attained by $\ell_{1}$-penalized regression (e.g. see Chernozhukov et al., 2018), in which case the desired rate holds if $s=o\left(\frac{\sqrt{n}}{\log\;\left(d\right)}\right)$.

Comparing the direct estimator from Section 4 with the approximation-based estimator here, when Assumption 5 holds with α=1, the requirements on the bias of Theorem 2 are essentially the same as those of Theorem 4, up to the rate condition on the $L_{2}$-norm instead of on the $L_{\infty }$-norm. In Hölder-smoothness models, this is a minor difference because estimation in $L_{\infty }$ typically yields the same convergence rate as estimation in $L_{2}$ up to a log factor (Tsybakov, 2009, Theorem 1.8).

Remark 1 It is natural to consider, assuming that the margin condition described in Assumption 5 holds, (a) whether and how the behaviour of the smooth approximation estimator ${\hat{L}}_{g}$ might improve, (b) whether and how to choose a sequence of tuning parameters $t_{n}$ in the LSE function (or some other smooth approximation to the pointwise maximum) to minimize the convergence rate and mean square error of ${\hat{L}}_{g_{t_{n}}}$ with respect to L(P), and (c) if we can describe minimax optimal estimators of L(P) under a large class of models (e.g. nuisance functions belonging to Hölder smooth classes). Careful analysis in a simplified setting (omitted here) reveals that while the approximation error $\left|L_{g_{t_{n}}}(P)-L(P)\right|$ improves from $O\left(\frac{1}{t_{n}}\right)$ to $O\left(\frac{1}{t_{n}^{1+\alpha }}\right)$ under a margin condition, the direct estimator dominates the smooth approximation even for optimally chosen data-adaptive tuning parameters $t_{n}$. Indeed, we conjecture that the direct estimator $\hat{L}$, modulo higher-order influence function corrections (J. Robins et al., 2008, 2009, 2017), is rate-optimal (up to log factors) when Assumption 5 holds. We leave precise minimax optimal rate characterization—for nonsmooth functionals similar to L(P) and U(P) under a margin condition akin to Assumption 5—to be investigated in future research.

### 5.4 Wald-type confidence interval for the ATE

Combining Theorem 1 with the smooth LSE-based approximation, we have


$$L_{g_{t}}(P)-\frac{\log\;8}{t}\leq E[Y(a=1)-Y(a=0)]\leq U_{h_{t}}(P)+\frac{\log\;8}{t}.$$

Thus, the interval


$$\left({\hat{L}}_{g_{t}}-\frac{\log\;8}{t}-z_{1-\delta /2}\sqrt{{\hat{V}}_{t}/n},\;{\hat{U}}_{h_{t}}+\frac{\log\;8}{t}+z_{1-\delta /2}\sqrt{{\hat{W}}_{t}/n}\right)$$

is an asymptotically valid (though potentially conservative) 100(1−δ)% Wald-type confidence interval for the ATE, where ${\hat{V}}_{t},{\hat{W}}_{t}$ are plug-in estimators of the nonparametric efficiency bounds ${\text{Var}}_{P}({\dot{L}}_{g_{t}}(O;P))$ and ${\text{Var}}_{P}({\dot{U}}_{h_{t}}(O;P))$, respectively. An ideal choice of the tuning parameter *t* requires balancing the smooth approximation error log8/t and the conditional bias term $E[{\hat{L}}_{g}|D^{n}]-L_{g}(P)$. Note that $\|\nabla^{2}g_{t}(\theta ){\|}_{\mathrm{op}}\leq t$ and Theorem 4 implies the conditional bias increases as we select a larger *t*. Hence, a smaller choice of *t* would reduce the conditional bias. On the other hand, reducing the approximation error log8/t requires a larger *t*. Combining approximation error of $L_{g_{t}}(P)$ with conditional bias and variance of ${\hat{L}}_{g_{t}}$, the conditional mean squared error (MSE) of ${\hat{L}}_{g_{t}}$ can be bounded as (note that the summand in ${\hat{L}}_{g_{t}}$ is bounded)


$$\begin{array}{rl} & E[({\hat{L}}_{g_{t}}-L(P){)}^{2}|D^{n}]\\ & \;≲\frac{1}{t^{2}}+\max_{y,a,z\in \{0,1\}}{\left\|{\hat{\pi }}_{ya.z}-\pi_{ya.z}\right\|}^{2}{\left\|{\hat{\lambda }}_{1}-\lambda_{1}\right\|}^{2}+t^{2}\max_{y,a,z\in \{0,1\}}{\left\|{\hat{\pi }}_{ya.z}-\pi_{ya.z}\right\|}^{4}+\frac{1}{n}, \end{array}$$

which leads to a putatively optimal choice of *t*, minimizing the conditional MSE, given by $t≍(\max_{y,a,z\in \{0,1\}}\|{\hat{\pi }}_{ya.z}-\pi_{ya.z}\|{)}^{-1}$. On its own, this is not a practical result since the error in estimating $\pi_{ya.z}$ is not known to the researcher. Moreover, the resulting rate of convergence under this choice of *t* is still first order in nuisance function error, which is not surprising given that the functional L(P) is nonsmooth (recall that we are not assuming a margin condition). How to choose *t* in a data-driven fashion to arrive at smallest estimation error of L(P) or the shortest confidence interval remain challenging open problems left for future investigation.

## 6 Direct estimation with continuous outcomes

Thus far, we have only focused on a binary outcome Y∈{0,1}. In this section, we will extend our approaches from the binary outcome case to construct valid bounds on the ATE for continuous outcomes. In fact, we will assume only that the outcome *Y* is bounded; without loss of generality, we may assume Y∈[0,1] (otherwise, one can always rescale the outcome). As observed but not studied in A. Balke and Pearl (1997), the idea is to replace the binary outcome in previous analyses with the indicator 1(Y≤s). Specifically, for each s∈[0,1], 1(Y≤s) is a binary outcome for which we can apply the bounds proposed in Section 3.1 to obtain pointwise bounds on the difference in the distribution functions of potential outcomes P(Y(a)≤s). We proceed by integrating the tail probabilities P(Y(a)>s) to bound the mean of potential outcomes E(Y(a)), and finally arrive at bounds on the ATE, E(Y(a=1)−Y(a=0)).

For any a,z∈{0,1} and s∈[0,1], define the probabilities $\pi_{1a.z}(s,X)=P(Y\leq s,A=a|X,Z=z)$ and $\pi_{0a.z}(s,X)=P(Y>s,A=a|X,Z=z)$. Next, we define functions exactly as in (1) and (2), replacing with $\pi_{ya.z}(X)$ with $\pi_{ya.z}(s,X)$ (note that $\theta_{u,j}$, $\theta_{\ell ,j}$, $\gamma_{\ell }$, and $\gamma_{u}$ are then also functions of *s* and X). Assuming *Z* is a valid instrument given covariates X, for each s∈[0,1] we view 1(Y≤s) as the binary outcome and apply the ‘Balke–Pearl’ bounds to this new outcome. By Theorem 1, we obtain the following bounds on the difference in distribution functions of the individual potential outcomes:


$$\gamma_{\ell }(s,X)\leq P[Y(a=1)\leq s|X]-P[Y(a=0)\leq s|X]\leq \gamma_{u}(s,X).$$

Integrating with respect to X and noting that $E(Y(a))=\int_{0}^{1}P[Y(a)>s]\;dt$, we arrive at the following valid (though not necessarily tight) bounds on the ATE:


(7) $$-\int_{0}^{1}E\left(\gamma_{u}(s,X)\right)ds\leq E(Y(a=1)-Y(a=0))\leq -\int_{0}^{1}E\left(\gamma_{\ell }(s,X)\right)ds.$$

One natural approach would be to estimate the functions $s\mapsto E(\gamma_{u}(s,X))$ and $s\mapsto E(\gamma_{\ell }(s,X))$ by the proposals of Section 4 or Section 5, over a grid of values s∈[0,1], and use these estimates to approximate the integrated bounds given in (7). Alternatively, one could use Monte Carlo methods to approximate the integral. Either approach would require computing a binary-outcome estimator at many different inputs *s*, which could be quite computationally intensive. The following theorem characterizes a looser bound that can be computed more efficiently.

Theorem 5 Let W∼Uniform(0,1) be independent of O=(X,Z,A,Y). Then

$$\begin{array}{rl} & \int_{0}^{1}E\left(\gamma_{u}(s,X)\right)ds\leq E\left[\min_{1\leq j\leq 8}{\tilde{\theta }}_{u,j}(X)\right],\\ & \int_{0}^{1}E\left(\gamma_{\ell }(s,X)\right)ds\geq E\left[\max_{1\leq j\leq 8}{\tilde{\theta }}_{\ell ,j}(X)\right], \end{array}$$

where we define ${\tilde{\pi }}_{1a.z}(X)=P(Y\leq W,A=a|X,Z=z),$  ${\tilde{\pi }}_{0a.z}(X)=$  P(Y>W,A=a∣X,Z=z), and where ${\tilde{\theta }}_{\ell ,j}(X)$ and ${\tilde{\theta }}_{u,j}(X)$ are defined exactly as in equations (1) and (2), replacing *π* with $\tilde{\pi }$.

Theorem 5 together with (7) implies the following valid bounds on the ATE:


(8) $$-E\left[\min_{1\leq j\leq 8}{\tilde{\theta }}_{u,j}(X)\right]\leq E[Y(1)-Y(0)]\leq -E\left[\max_{1\leq j\leq 8}{\tilde{\theta }}_{\ell ,j}(X)\right].$$

Note that the bounds in (8) are of the same form as those in Theorem 1, but with a new binary outcome 1(Y≤W). By introducing an independent uniform random variable, we obtain a looser bound, but one that lends itself to more computationally feasible estimation. To operationalize these bounds, we can simulate *n* i.i.d. Uniform(0,1) random variables $W_{1},\ldots ,W_{n}$, independent of the data, and construct the augmented observation unit $\tilde{O}=(X,Z,A,Y,W)$. We then estimate the quantities in the bounds exactly as in the binary case using the methods proposed in Section 4 or Section 5, with the new binary outcome being 1(Y≤W). Note that the exact value of the final bounds will depend on the realization of $W_{1},\ldots ,W_{n}$. To reduce this variability and regain some efficiency, we may repeat this process *m* times and average the resulting estimates of the bounds—see online supplementary material, Appendix S5 for an illustration of this behaviour in a simplified setting.

## 7 Simulation studies

In order to assess the performance of the proposed estimators, we conducted two simulation studies. For brevity, we provide full details of data-generating mechanisms and results in online supplementary material, Appendix S6, and only summarize the main points here.

In the first simulation study, we use a complex form for the data-generating process, under which we compare methods for the proposed estimators $\left(\hat{L},{\hat{L}}_{g_{t}},\hat{U},{\hat{U}}_{g_{t}}\right)$. Specifically, we compare the use of flexible ensembles for estimating the nuisance functions to (i) naïve unadjusted Balke–Pearl bounds and (ii) parametric models for the nuisance functions. We find that our proposed flexible approaches exhibit very little bias and reasonable standard errors. On the other hand, unadjusted bounds are much wider, and parametric estimators for the nuisance functions result in a substantial degree of bias for the true adjusted bounds.

In the second simulation study, we consider a simplified scenario where Assumption 5 holds with margin parameter α=1, and demonstrate for different sample sizes *n* how mean squared errors of the proposed lower bound estimators $\hat{L}$ and ${\hat{L}}_{g_{t}}$ compare to that of a plug-in estimator. We find that, as anticipated by our theoretical results, our proposed estimators generally have smaller error, and achieve parametric levels of error when the root-mean-square error of nuisance estimates is less than $O(n^{-1/4})$.

## 8 Data analysis

To illustrate the proposed methods on real data, we aimed to estimate the effect of higher education on wages later in life using a subset of data from the National Longitudinal Survey of Young Men (Card, 1995), as previously analysed, for instance, in Tan (2006), L. Wang and Tchetgen Tchetgen (2018). For ease of comparison, we replicate the setup of L. Wang and Tchetgen Tchetgen (2018), and use the n=3,010 participants (males between 14 and 24 years in 1966) for whom survey responses for education and wage in 1976 were available. In particular, the instrument *Z* is taken to be an indicator of proximity to a 4-year college, the exposure *A* an indicator of education beyond high school, and the outcome *Y* an indicator of wages in 1976 exceeding the median value. In addition to (Z,A,Y), we adjusted for a collection of baseline covariates X: age, parental educational attainment, indicators for living in the south and a metropolitan area, race, intelligence quotient scores, as well as variable-specific missingness indicators. As in Card (1995) and L. Wang and Tchetgen Tchetgen (2018), mean imputation was used for missing covariates, and, due to nonrepresentativeness of the sample, the observations were reweighted using sampling weights (results from unweighted analyses were qualitatively similar).

L. Wang and Tchetgen Tchetgen (2018) consider the binary IV problem (i.e. Z,A,Y∈{0,1}) as we do, and propose several estimators that respect that the CATE and ATE must be bounded between −1 and 1. Note that, in addition to the assumptions adopted here which underpin the Balke–Pearl bounds, these authors introduced a structural assumption—asserting no additive interaction between unmeasured confounders and either (i) the instrument, on the treatment, or (ii) the treatment, on the outcome—which results in point identification of the CATE and thus the population ATE. That is, their methods rely on strictly stronger assumptions than ours.

We computed estimates of the covariate-adjusted bounds L(P) and U(P) using the direct estimators $\hat{L}$ and $\hat{U}$ developed in Section 4, assuming the margin condition (i.e. Assumption 5). Specifically, we used separate ensembles based on fits from glm, rpart, ranger, and polymars using the SuperLearner package in R (Polley et al., 2019) to obtain ${\hat{\lambda }}_{1}(X)$ and $\left\{{\hat{\pi }}_{ya.z}(X)\;:\;y,a,z\in \{0,1\}\right\}$. We also computed LSE-based estimators ${\hat{L}}_{g_{t}}$ and ${\hat{U}}_{h_{t}}$ based on the same nuisance function estimators, as described in Section 5, with the *ad hoc* tuning parameter choice $t=100n^{1/4}$. For all estimators, we used cross-fitting with five folds.

Summarizing the results, the point estimates for the covariate-adjusted Balke–Pearl bound estimators $\hat{L}$ and $\hat{U}$ were (−0.405,0.546), and the resulting Wald-based 95% confidence interval for the ATE was (−0.454,0.597). These results indicate a wide range of possible effects compatible with the observed data, with the width perhaps demonstrating the potentially strong effects of unmeasured confounders. Nevertheless, quite surprisingly, compared to the bounds reported in L. Wang and Tchetgen Tchetgen (2018), the confidence interval width is narrower and the estimated upper bound is more informative. For instance, the point estimates and 95% confidence intervals for their proposed ‘*g*-estimator’ and ‘bounded multiply robust estimator’ were 0.079 (−0.355,1.000) and 0.344 (−0.373,0.938), respectively. This discrepancy may arise as their estimators rely on unstable weights which do not feed into our approach (i.e. the nonparametric efficiency bounds for the Balke–Pearl bound functionals are much less than that for the functional targeted by these authors), or because the constraints implied by the binary IV model are not explicitly incorporated in their approaches (cf. Proposition 1 in L. Wang & Tchetgen Tchetgen, 2018).

The approximation-based estimators ${\hat{L}}_{g_{t}}$ and ${\hat{U}}_{h_{t}}$ were nearly identical, (−0.406,0.544), and the associated 95% confidence interval constructed as in Section 5.4 was (−0.506,0.646): the extra width is due to the inclusion of the (potentially conservative) error bound log(8)/t.

## 9 Discussion

In this paper, we proposed estimators of nonparametric bounds on the ATE using an IV, avoiding strong structural or parametric assumptions typically used for point identification. We extended the classic approach of A. Balke and Pearl (1997) by incorporating baseline covariate information to (i) control for measured confounders to render the instrument valid, and/or (ii) construct narrower and hence more informative bounds. We also proposed a concrete extension to bound the ATE for a general bounded outcome. Our estimators are based on influence functions, and as a result are robust and can attain $\sqrt{n}$-consistency, asymptotic normality, and nonparametric efficiency, under nonparametric convergence rates of the component nuisance functions estimation—these rates are attainable under sparsity, smoothness or other structural conditions.

The key difficulty in estimating the covariate-adjusted bound functionals L(P) and U(P) is that, as means of nondifferentiable functions, they are not pathwise differentiable (Bickel et al., 1993). As a result, these parameters do not—at least without further assumptions—have an influence function to facilitate flexible and efficient estimation (e.g. see Section 10.5.3 of Kennedy, 2024). To make progress, in Section 4, we first proposed and invoked a margin condition to render the exact bound functionals pathwise differentiable, and proposed influence function-based estimators under this condition. Second, in Section 5, we presented general valid bounds on the ATE via estimation of pathwise differentiable approximations to L(P) and U(P). The second approach has the advantage of not requiring the margin condition, but the resulting bounds suffer from being slightly conservative, and not converging to the true exact bounds at fast rates. The first approach, on the other hand, can achieve parametric rates when the margin condition holds. As a general guideline, we recommend that practitioners use the direct-bound estimators proposed in Section 4, deferring to the approximate bounds only when there is serious doubt on Assumption 5 arising due to subject matter knowledge.

In our view, it is difficult to imagine concrete scenarios in which Assumption 5 would be violated. A similar margin condition is invoked in dynamic treatment regime problems, for example, when estimating the mean outcome under the optimal treatment policy in a point treatment setting: E[max{E(Y(a=1)∣X),E(Y(a=0)∣X)}] (Luedtke & van der Laan, 2016). In that context, one must argue that the CATE is well separated from zero, the value representing no treatment effect. It is plausible that there is a subgroup for which the CATE is exactly zero, which would violate the margin condition for that problem. In our setting, Assumption 5 requires separation of the pointwise maximum (minimum) of the Balke–Pearl lower (upper) bound functions $\theta_{\ell }(X)$ ($\theta_{u}(X)$) from the second largest (smallest) value. These lower and upper bound functions are not readily interpretable like the CATE, and it would seem to necessitate an unlikely confluence of factors to violate the required separation.

The approaches developed in this paper are not inherently tied to the specific bounds we studied: the same ideas extend to many functionals defined as expectations of nonsmooth functions, which arise often in problems targeting bounds on causal effects and beyond, e.g. in change point detection problems (J. Wang et al., 2023). Thus, in online supplementary material, Appendix S1, we frame the proposed methods more generally for a large class of nonsmooth functionals. Indeed, one month after our work was posted on arXiv, Semenova (2023) independently considered estimation of bounds, and functionals thereof, that can be expressed as the minimum of several nuisance functions. They studied a wide range of bounds obtained from certain optimization problems and proposed estimators under a margin condition similar to Assumption 5. The Balke–Pearl bounds arise from a linear programming specification, which is a special case of bounds based on general optimization problems in Semenova (2023). However, our work specifically characterizes the nonparametric efficient estimator of the Balke–Pearl bounds, as well as its asymptotic bias, in Theorem 2. Further, a direct application of the approach of Semenova (2023) to our setting would require that instrument probabilities $\lambda_{1}(X)$ are known. We also propose estimators based on smooth approximation—not considered in Semenova (2023)—and establish corresponding efficiency theory. We expect that the methods discussed in this paper can be combined with the insights from Semenova (2023) to yield efficient, debiased estimators in more general settings as considered in their paper.

It is important to mention some limitations of the proposed methods, as well as possible extensions and open problems. First, we have throughout assumed a fixed collection of measured covariates X. In Proposition 1, we provide a general criterion to justify adjusting for certain covariates, however, it remains to characterize (i) the actual difference in the length of the bounds based on two valid adjustment sets, and (ii) the effect of the adjustment set on the variance of the proposed estimators. One option for covariate selection, proposed by an anonymous reviewer, could proceed by assessing changes in bound length over a priority-based nesting of covariate subsets, extending recent work in stability-based, change-in-estimate covariate selection for ATE estimation (Keele & Small, 2021; Loh & Vansteelandt, 2021). Second, we have focused primarily on the setting with instrument, exposure, and outcome all binary variables, since this is the simplest setting for partial identification with closed-form bounds given by A. Balke and Pearl (1997). In Section 6, we provided an extension to construct valid ATE bounds for continuous outcomes, though an important open problem is determining the sharpest possible bounds for such general outcomes, beyond the binary case. Along the same lines, it of course will also be of interest to consider multi-valued or continuous instruments and exposures, as well as time-varying IV settings which arise frequently in practice (e.g. in bidding problems). In these cases, there is generally no longer the same closed-form solution for the theoretical bounds, and alternative optimization specifications and/or symbolic bounds might be incorporated (Duarte et al., 2023; Sachs et al., 2022; Zhang & Bareinboim, 2021). When closed-form solutions are available (see examples in Sachs et al., 2022), similar direct estimation and targeting smooth approximation strategies can be applied to estimate the bounds efficiently (see online supplementary material, Appendix S1 for more discussion). Duarte et al. (2023) recently proposed a general framework to automatically compute sharp bounds on causal effects under user-specified causal assumptions. Their idea is to formalize the assumptions and observed evidence as polynomial constraints, and estimands of interest as objective functions, following which the bounds are obtained by optimizing the objective. In this regard, the Balke–Pearl bounds can be viewed as a special case where the causal assumptions are specified by an IV model. However, the Balke–Pearl bounds admit closed-form solutions, based on which efficiency estimation theory can be derived, while there is no closed-form solutions to the general optimization problem in Duarte et al. (2023). Moreover, they only focus on the fully discrete setting since the optimization problem is intractable in greater generality, while the methodology in our work naturally handles continuous covariates (and outcomes using the extension in Section 6). Third, while our results do not depend on any strength of association between *Z* and *A*, weak instruments are not ideal for the purposes of partial identification: Balke–Pearl bounds are wider and less informative for weaker instruments. An advantage of our covariate-adjusted approach is that more informative sharp bounds on the CATE may be possible for some strata (e.g. for which the instrument is less weak), which could be missed by a marginal analysis. Fourth, and lastly, an interesting problem we will explore in future research is to directly and efficiently estimate the conditional bounds $\gamma_{\ell }(X)$, $\gamma_{u}(X)$ for the CATE given in Theorem 1, and identify strata for whom we are confident that the CATE is positive or negative. In doing so, one may extend the ideas in Kennedy et al. (2020) to define ‘sharpness’ of an instrument—the ability to tightly bound causal effects in certain subgroups—without relying on the typical monotonicity assumption.

## Supplementary Material

qkaf028_Supplementary_Data

## Data Availability

The code for reproducing the motivating illustration, simulation studies, and data application are available at https://github.com/alexlevis/cov-assisted-bounds-JRSSB. The data for the application are freely available online, and references are provided in the above code repository.
