# Supplementary material for: Covariate-assisted bounds on causal effects with instrumental variables
Source: J R Stat Soc Series B Stat Methodol. 2025 May 27;87(5):1508–27. doi: 10.1093/jrsssb/qkaf028 (PMC12602419; doi:10.1093/jrsssb/qkaf028)
Supplement: qkaf028_Supplementary_Data [file qkaf028_supplementary_data.pdf]

Supplement to "Covariate-assisted bounds on causal effects with instrumental variables"

Alexander W. Levis[1], Matteo Bonvini[1], Zhenghao Zeng[1],
Luke Keele[2], Edward H. Kennedy[1]

[1]Department of Statistics & Data Science,
Carnegie Mellon University
[2]Department of Surgery,
University of Pennsylvania

{alevis, mbonvini, zhenghaz} @ andrew.cmu.edu;
luke.keele@gmail.com; edward@stat.cmu.edu

S1 Strategies for Robust Non-smooth Functional Estimation

In this work we focus on the estimation of bounds on causal effects, which results in an interesting example of non-smooth functional estimation. Mathematically, a large class of such functionals are of the form $\mathbb{E}[g(\theta(\boldsymbol{X}))]$, where $\theta : \mathcal{X} \mapsto \mathbb{R}^d$ is some task-dependent function of the covariates, and $g : \mathbb{R}^d \mapsto \mathbb{R}$ is a continuous function but not differentiable on a subset of $\mathbb{R}^d$. In the covariate-assisted Balke-Pearl bounds example (i.e., $\mathbb{E}[\max_{1\leq j\leq 8} \theta_{\ell,j}(\boldsymbol{X})]$), $\theta = \theta_\ell$ is defined in (1) and g is the max function. The non-differentiability of g renders the target functional non-smooth and hence influence-function-based estimation procedures cannot be applied directly. Non-smooth functionals in this form are very common in the literature (Makarov, 1982; Luedtke and van der Laan, 2016; Fan et al., 2017; Semenova, 2023) and we outline some general strategies to estimate them using ideas explored in our work.

S1.1 Targeting Smooth Approximation

The first idea is an intuitive one, in which we approximate the non-differentiable function with a smooth approximation, and target this approximation in a debiased manner. This relates to ideas from the non-smooth optimization literature. In seminal work, Nesterov (2005) proposed to approximate a non-smooth objective with a smooth function and optimize the smooth approximation instead. When the objective function is smooth, efficient optimization algorithms such as Nesterov's accelerated method could be applied to achieve fast convergence rates. The idea is similar in non-smooth functional estimation: we replace the non-smooth function g with its approximation in $\mathbb{E}[g(\theta(\boldsymbol{X}))]$ and target the smooth approximation instead. Then we construct an estimator of this smooth functional using its influence function, yielding fast statistical rates of convergence. An important trade-off to keep in mind, however, is that there is additional error due to the approximation step that contributes to the mean-squared error, say, in estimation of the original target functional.

To formalize this approach, suppose the family of functions $\{g_t : t \in (0, \infty)\}$ approximates g, such that $g_t \to g$ pointwise as $t \to \infty$. Moreover, define $\chi = \mathbb{E}(g(\theta(\boldsymbol{X})))$, and suppose g_t

is sufficiently smooth such that the functional $\chi_t = \mathbb{E}(g_t(\theta(\boldsymbol{X})))$ is pathwise differentiable for each $t \in (0, \infty)$.

Example 1 (Value of the optimal treatment regime)**.** Let $O = (\boldsymbol{X}, A, Y) \in \mathbb{R}^d \times \{0,1\} \times \mathbb{R}$, let $\mu(\boldsymbol{X}, a) = \mathbb{E}(Y \mid \boldsymbol{X}, A = a)$ for $a \in \{0,1\}$, and consider the functional

$$\chi = \mathbb{E}(\mathbb{1}(\tau(\boldsymbol{X}) > 0)\tau(\boldsymbol{X})) = \mathbb{E}(\max\{\tau(\boldsymbol{X}), 0\}),$$

where $\tau(\boldsymbol{X}) = \mu(\boldsymbol{X}, 1) - \mu(\boldsymbol{X}, 0)$. This is related to the value of the optimal treatment regime which assigns treatment $A = 1$ when $\tau > 0$, and otherwise $A = 0$ (Murphy, 2003). This fundamental non-smooth functional was studied in Luedtke and van der Laan (2016), and falls into our framework where $\theta(\boldsymbol{X}) \equiv (\mu(\boldsymbol{X}, 0), \mu(\boldsymbol{X}, 1))^T$, and $g(\theta_1, \theta_2) = \max\{\theta_2 - \theta_1, 0\}$. One natural series of approximations is $\{g_t : t \in (0, \infty)\}$ where $g_t(\theta_1, \theta_2) = \frac{1}{t}\log\left(1 + e^{t(\theta_2 - \theta_1)}\right)$.

The approximation g_t in Example 1 is an instance of log-sum-exp, exactly as employed in Section 5 for the Balke-Pearl bounds. While this simple example fits precisely within the initial framing described in the beginning of this appendix (i.e., the mean of a non-differentiable but continuous function of nuisance functions), note that the smooth approximation approach can be applied in a much broader class of non-smooth problems; consider the following more challenging example.

Example 2 (Dose-response curve)**.** Let $O = (\boldsymbol{X}, A, Y) \in \mathbb{R}^d \times \mathbb{R} \times \mathbb{R}$, where A now is a continuous treatment, and let $\mu(\boldsymbol{X}, a) = \mathbb{E}(Y \mid \boldsymbol{X}, A = a)$ for $a \in \mathbb{R}$. Under standard causal assumptions, $\chi = \mathbb{E}(\mu(\boldsymbol{X}, a_0))$ represents the dose-response curve at a_0, $\mathbb{E}(Y(a_0))$, i.e., the mean of the potential outcome under an intervention setting treatment to $A = a_0$. In this case, one can consider the nuisance functions $\{\mu(\boldsymbol{X}, a) : a \in \mathbb{R}\}$ to lie in an infinite dimensional space, with g picking out one component. In any case, the functional χ is not pathwise differentiable, and Bibaut and van der Laan (2017) and Branson et al. (2023) replace χ with a smooth approximation

$$\chi_t = \mathbb{E}\left(\int K_{1/t}(a - a_0)\mu(\boldsymbol{X}, a)\, da\right),$$

for some smooth symmetric kernel function $K_{1/t}(\,\cdot\,)$ with bandwidth parameter $1/t$. Here, the "selection" function g is approximated by the kernel-smooth function $g_t(\{\mu(\boldsymbol{X}, a) : a \in \mathbb{R}\}) = \int K_{1/t}(a - a_0)\mu(\boldsymbol{X}, a)\, da$ which averages $\mu(\boldsymbol{X}, a)$ in a neighborhood around a_0.

Once the smooth approximation functional χ_t is established, one can proceed with a debiased estimator based on the influence function of χ_t (see Appendix S4.1 for an overview of this set of ideas). In Example 1, one can employ the one-step estimator

$$\widehat{\chi}_t = \mathbb{P}_n\left[\frac{1}{t}\log\left(1 + e^{t\widehat{\tau}(\boldsymbol{X})}\right) + \frac{e^{t\widehat{\tau}(\boldsymbol{X})}}{1 + e^{t\widehat{\tau}(\boldsymbol{X})}}\left\{\frac{A}{\widehat{\eta}(\boldsymbol{X})} - \frac{1 - A}{1 - \widehat{\eta}(\boldsymbol{X})}\right\}(Y - \widehat{\mu}(\boldsymbol{X}, A))\right],$$

where $\eta(\boldsymbol{X}) = \mathbb{P}[A = 1 \mid X]$, while in Example 2 one can use

$$\widehat{\chi}_t = \mathbb{P}_n\left[K_{1/t}(A - a_0)\frac{Y - \widehat{\mu}(\boldsymbol{X}, A)}{\widehat{\pi}(A \mid \boldsymbol{X})} + \int K_{1/t}(a - a_0)\widehat{\mu}(\boldsymbol{X}, a)\, da\right],$$

where $\pi(a \mid \boldsymbol{X})$ is the density of A given $\boldsymbol{X}$ at $a \in \mathbb{R}$. For a fixed t, just as in Theorem 4 for the approximated Balke-Pearl bounds, one can then establish the convergence properties of $\widehat{\chi}_t$ with respect to its smooth target χ_t—under mild conditions,

$$\widehat{\chi}_t - \chi_t = \mathbb{P}_n\phi_t(O) + O_{\mathbb{P}}(R_n) + o_{\mathbb{P}}(n^{-1/2}),$$

where ϕ_t is the influence function of χ_t, and R_n is a second-order asymptotic bias. In Example 1, $R_n = \|\widehat{\eta}-\eta\| \cdot \|\widehat{\mu}-\mu\| + \|\widehat{\mu}-\mu\|^2$, where $\|\widehat{\mu}-\mu\| = \max_{a\in\{0,1\}} \|\widehat{\mu}(\cdot, a)-\mu(\cdot, a)\|$, whereas in Example 2, $R_n = \sup_{\boldsymbol{x},a} \sqrt{\mathbb{E}(\{\widehat{\mu}(\boldsymbol{x},a) - \mu(\boldsymbol{x},a)\}^2)} \cdot \sup_{\boldsymbol{x},a} \sqrt{\mathbb{E}(\{\widehat{\pi}(a \mid \boldsymbol{x}) - \pi(a \mid \boldsymbol{x})\}^2)}$. That is, the rate at which $\widehat{\chi}_t$ converges to χ_t depends on the product of nuisance function errors; in this sense, the approximation estimator exhibits *fast* convergence to its smooth target.

As alluded to earlier, the matter of convergence of $\widehat{\chi}_t$ to the *original* non-smooth target functional χ—say, for some sequence of tuning parameters $t \equiv t_n \to \infty$— is more complicated. Consider the simple error decomposition $\widehat{\chi}_t - \chi = (\widehat{\chi}_t - \chi_t) + (\chi_t - \chi)$. While we have analyzed the first term, the approximation error term is fundamental to this question. In Example 1, for instance, $|\chi_t - \chi| \leq \frac{\log(2)}{t}$. However, simply taking $t \equiv \infty$ may not be optimal (unless a margin condition holds, as explored in the following section), as the smooth error term $\widehat{\chi}_t - \chi_t$ must be more carefully analyzed to acknowledge the growing sequence of tuning parameters $t \equiv t_n$. More careful analysis of the asymptotic bias term R_n in Example 1 reveals the term $t \cdot \|\widehat{\mu} - \mu\|^2$. An optimal choice of t must then balance the contributions to the approximation error, and the smooth asymptotic bias. These convergence questions are deep and of great interest, and will be further pursued in future research.

S1.2 Estimation Under a Margin Condition

In the case where g is the max function $g(\mathbf{x}) = \max_{1\leq i\leq d} x_i$ (Qian and Murphy, 2011; Luedtke and van der Laan, 2016; Sachs et al., 2022), one could impose a margin condition, often used in classification literature to derive sharp bounds on excess risk (Audibert and Tsybakov, 2007), to obtain an estimator with a higher-order bias term. Considering a generic target functional $\mathbb{E}[\max_{j\in[d]} \theta_j(\boldsymbol{X})]$, a margin condition is summarized in the following assumption.

Assumption 1. *(Margin condition) There exists a constant* $\alpha \geq 0$ *such that*

$$\mathbb{P}\left(\max_{j\in[d]} \theta_j(\boldsymbol{X}) - \max_{j\notin \arg\max_{j\in[d]} \theta_j(\boldsymbol{X})} \theta_j(\boldsymbol{X}) \leq t\right) \lesssim t^\alpha \quad \forall t > 0.$$

Here $\max_{j\in[d]} \theta_j(\mathbf{x}) - \max_{j\notin \arg\max_{j\in[d]} \theta_j(\mathbf{x})} \theta_j(\mathbf{x})$ can be interpreted as the "margin" of θ at observation $\boldsymbol{X} = \mathbf{x}$ and quantifies the difference between the maximum and the second-largest θ_j function evaluated at $\mathbf{x}$. Thus the margin condition bounds the probability of an event on which the maximum and the second-largest θ_j function are close, in which case it would be hard to distinguish between them. Intuitively, this event characterizes observations that are close to the non-differentiable set of max function and makes it difficult to find the right maximizer and perform estimation. Imposing a bound on this "difficult" event could then yield favorable situations where small estimation error is possible. Apart from the covariate-assisted Balke-Pearl bounds mainly considered in this work, we provide two additional examples below, for which margin conditions could be applied.

Example 3. (Frechet-Hoeffding bounds) Suppose we are interested in the proportion of subjects of the type "always-recover", $\mathbb{P}(Y(1) = Y(0) = 1)$, for a binary outcome under an exchangeability assumption. The bounds on $\mathbb{P}(Y(1) = Y(0) = 1)$ are given by

$$\mathbb{E}[\max(\mu(\boldsymbol{X}, 0) + \mu(\boldsymbol{X}, 1) - 1, 0)] \leq \mathbb{P}(Y(1) = Y(0) = 1) \leq \mathbb{E}[\min(\mu(\boldsymbol{X}, 0), \mu(\boldsymbol{X}, 1))],$$

where $\mu(\mathbf{x}, a) = \mathbb{E}[Y \mid \boldsymbol{X} = \mathbf{x}, A = a]$.

Example 4. (Measurement error example in Sachs et al. (2022)) Suppose we have a binary variable A affecting a binary outcome Y, but Y is not observed. Instead, the binary variable Y' which is a child of Y, is observed, with the effect of Y on the measured Y' confounded. Under monotonicity $Y'(Y=1) \geq Y'(Y=0)$, we have

$$\begin{aligned} \mathbb{E}[Y(1)] - \mathbb{E}[Y(0)] \in [&\max\{-1, 2\mathbb{P}(Y'=0 \mid A=0) - 2\mathbb{P}(Y'=0 \mid A=1) - 1\} \\ &\min\{1, 2\mathbb{P}(Y'=0 \mid A=0) - 2\mathbb{P}(Y'=0 \mid A=1) + 1\}]. \end{aligned} \tag{1}$$

Though the original bounds do not involve covariates (so treatment is randomly assigned), one could adjust for covariates by assuming exchangeability to incorporate covariates in (1) and obtain similar covariate-assisted bounds as we mainly consider in the main text.

The "direct" estimation strategy is then re-writing target functional as

$$\mathbb{E}[\max_{j\in[d]} \theta_j(\boldsymbol{X})] = \sum_{j=1}^{d} \mathbb{E}\left[\mathbb{1}\left(j = \arg\max_{k\in[d]} \theta_k(\boldsymbol{X})\right)\theta_j(\boldsymbol{X})\right]$$

and derive its influence function and corresponding efficiency theory while viewing the indicator $\mathbb{1}\left(j = \arg\max_{k\in[d]} \theta_k(\boldsymbol{X})\right)$ as a known function. To derive the doubly robust estimator based on influence functions, one could plug-in an "estimate" of the indicator as $\mathbb{1}\left(j = \arg\max_{k\in[d]} \hat{\theta}_k(\boldsymbol{X})\right)$ with $\hat{\theta}_k$ being an initial estimator of θ_k. Under the margin condition, one could expect smaller conditional bias of doubly robust estimator, following proof techniques of Theorem 2.

As we point out in the main text, there is a trade-off on which method to use when g is the max function. Smooth approximation requires choosing a tuning parameter in the approximation function while the direct approach requires researchers' belief in the margin condition. In general we recommend the direct approach when there is no clear evidence that the margin condition is violated.

We now consider a generalization of the above approach to parameters of the form

$$\psi = \mathbb{E}\left(s\left[g_1(\theta(\mathbf{X}))\mathbb{1}\{f_1(\theta(\mathbf{X}) < 0\}, \ldots, g_K(\theta(\mathbf{X}))\mathbb{1}\{f_K(\theta(\mathbf{X})) < 0\}\right]\right), \text{ where}$$

$s : \mathbb{R}^K \to \mathbb{R}$, $g_k : \mathbb{R}^d \to \mathbb{R}$ and $f_k : \mathbb{R}^d \to \mathbb{R}$ are known smooth functions and $\theta : \mathcal{X} \mapsto \mathbb{R}^d$ is a function that needs to be learned from the data. For example, let $\psi = \mathbb{E}[\max\{\theta_1(\mathbf{X}), \theta_2(\mathbf{X}), \theta_3(\mathbf{X})\}]$. We can express this functional as

$$\begin{aligned} \psi = &\mathbb{E}\left[\theta_1(\mathbf{X})\mathbb{1}\{\theta_2(\mathbf{X}) - \theta_1(\mathbf{X}) < 0\} \cdot \mathbb{1}\{\theta_3(\mathbf{X}) - \theta_1(\mathbf{X}) < 0\}\right] \\ &+ \mathbb{E}\left[\theta_2(\mathbf{X})\mathbb{1}\{\theta_1(\mathbf{X}) - \theta_2(\mathbf{X}) < 0\} \cdot \mathbb{1}\{\theta_3(\mathbf{X}) - \theta_2(\mathbf{X}) < 0\}\right] \\ &+ \mathbb{E}\left[\theta_3(\mathbf{X})\mathbb{1}\{\theta_1(\mathbf{X}) - \theta_3(\mathbf{X}) < 0\} \cdot \mathbb{1}\{\theta_2(\mathbf{X}) - \theta_3(\mathbf{X}) < 0\}\right]. \end{aligned}$$

We can thus take $g_1(\theta(\mathbf{X})) = \theta_1(\mathbf{X})$, $f_1(\theta(\mathbf{x})) = \theta_2(\mathbf{X}) - \theta_1(\mathbf{X})$, $g_2(\theta(\mathbf{X})) = 1$, $f_2(\theta(\mathbf{X})) = \theta_3(\mathbf{X}) - \theta_1(\mathbf{X})$, and so on. Then, we can take

$$s(t_1, t_2, t_3, t_4, t_5, t_6) = t_1 t_2 + t_3 t_4 + t_5 t_6.$$

Let $\lambda_k = \mathbb{1}\{f_k(\theta(\mathbf{x})) < 0\}$ and $\widehat{\lambda}_k = \mathbb{1}\{f_k(\widehat{\theta}(\mathbf{x}) < 0\}$ its estimator. Further, let the uncentered influence function of the parameter ψ be denoted by $\nu(O; \eta, \lambda_1, \ldots, \lambda_K)$, treating each λ_k as

known, and where η denotes all other nuisance functions. One of the two estimators of ψ that we study in this work is

$$\widehat{\psi} = \frac{1}{n}\sum_{i=1}^{n} \nu(O_i; \widehat{\eta}, \widehat{\lambda}_1, \dots, \widehat{\lambda}_K)$$

We can decompose conditional bias in two parts:

$$\begin{aligned}
&\widehat{\psi} - \psi \\
&= \mathbb{E}\left(\nu(O; \widehat{\eta}, \widehat{\lambda}_1, \dots, \widehat{\lambda}_K) - s\left[g_1(\theta(\mathbf{X}))\mathbb{1}\{f_1(\widehat{\theta}(\mathbf{X}) < 0\}, \dots, g_K(\theta(\mathbf{X}))\mathbb{1}\{f_K(\widehat{\theta}(\mathbf{X})) < 0\}\right] \mid D^n\right) \\
&\quad + \mathbb{E}\left(s\left[g_1(\theta(\mathbf{X}))\mathbb{1}\{f_1(\widehat{\theta}(\mathbf{X}) < 0\}, \dots, g_K(\theta(\mathbf{X}))\mathbb{1}\{f_K(\widehat{\theta}(\mathbf{X})) < 0\}\right] \mid D^n\right) \\
&\qquad - \mathbb{E}\left(s\left[g_1(\theta(\mathbf{X}))\mathbb{1}\{f_1(\theta(\mathbf{X}) < 0\}, \dots, g_K(\theta(\mathbf{X}))\mathbb{1}\{f_K(\theta(\mathbf{X})) < 0\}\right] \mid D^n\right)
\end{aligned}$$

The first term does not involve any non-smooth transformation of $\mathbb{P}$ that is unknown; it is thus easier to control and, in virtue of the use of the first-order IF, it should have second-order nuisance bias. If s is Lipschitz, then the second term can be upper bounded by a constant multiple of

$$\begin{aligned}
&\mathbb{E}\left(\sqrt{\sum_{k=1}^{K} g_k^2(\theta(\mathbf{X}))\left[\mathbb{1}\{f_k(\widehat{\theta}(\mathbf{X}) < 0\} - \mathbb{1}\{f_k(\theta(\mathbf{X}) < 0\}\right]^2} \mid D^n\right) \\
&\leq \max_{1\leq k\leq K} \mathbb{E}\left[|g_k(\theta(\mathbf{X}))|\left|\mathbb{1}\{f_k(\widehat{\theta}(\mathbf{X}) < 0\} - \mathbb{1}\{f_k(\theta(\mathbf{X}) < 0\}\right| \mid D^n\right] \\
&\leq \max_{1\leq k\leq K} \mathbb{E}\left[|g_k(\theta(\mathbf{X}))|\,\mathbb{1}\left\{|f_k(\theta(\mathbf{X})| \leq \left|f_k(\widehat{\theta}(\mathbf{X})) - f_k(\theta(\mathbf{X}))\right|\right\} \mid D^n\right] \\
&\leq \max_{1\leq k\leq K}\left[\mathbb{E}\left\{\frac{g_k^2(\theta(\mathbf{X}))}{f_k^2(\theta(\mathbf{X}))} \mid D^n\right\}\right]^{1/2} \\
&\qquad \times \left(\mathbb{E}\left[f_k^2(\theta(\mathbf{X}))\mathbb{1}\left\{|f_k(\theta(\mathbf{X})| \leq \left|f_k(\widehat{\theta}(\mathbf{X})) - f_k(\theta(\mathbf{X}))\right|\right\} \mid D^n\right]\right)^{1/2} \\
&\leq \max_{1\leq k\leq K}\left[\mathbb{E}\left\{\frac{g_k^2(\theta(\mathbf{X}))}{f_k^2(\theta(\mathbf{X}))} \mid D^n\right\}\right]^{1/2} \\
&\qquad \times \max_{1\leq k\leq K}\sup_{\mathbf{x}\in\mathcal{X}}\left|f_k(\widehat{\theta}(\mathbf{x})) - f_k(\theta(\mathbf{x}))\right| \mathbb{P}\left(|f_k(\theta(\mathbf{X}))| \leq \sup_{\mathbf{x}\in\mathcal{X}}\left|f_k(\widehat{\theta}(\mathbf{x})) - f_k(\theta(\mathbf{x}))\right| \mid D^n\right)
\end{aligned}$$

Notice that we also have the bound

$$\begin{aligned}
&\max_{1\leq k\leq K} \mathbb{E}\left[|g_k(\theta(\mathbf{X}))|\left|\mathbb{1}\{f_k(\widehat{\theta}(\mathbf{X}) < 0\} - \mathbb{1}\{f_k(\theta(\mathbf{X}) < 0\}\right| \mid D^n\right] \\
&\leq \max_{1\leq k\leq K}\left[\mathbb{E}\left\{g_k^2(\theta(\mathbf{X})) \mid D^n\right\}\right]^{1/2} \mathbb{P}\left(|f_k(\theta(\mathbf{X}))| \leq \sup_{\mathbf{x}\in\mathcal{X}}\left|f_k(\widehat{\theta}(\mathbf{x})) - f_k(\theta(\mathbf{x}))\right| \mid D^n\right).
\end{aligned}$$

So we can take the minimum between the two bounds as the final bound. The second bound would be useful, for instance, if, for some k, $\mathbb{P}(f_k(\theta(\mathbf{X})) = 0) > \epsilon$, in which case the margin condition would not hold. Finally, if we directly apply this bound to the example $\psi = \mathbb{E}[\max\{\theta_1(\mathbf{X}), \theta_2(\mathbf{X}), \theta_3(\mathbf{X})\}]$, we would obtain a bound that would match the order of the one from the main text if the margin condition $\mathbb{P}\left(|\theta_k(\mathbf{X}) - \theta_s(\mathbf{X})| \leq t\right) \lesssim t^\alpha$ holds for every $k, s \in \{1, \dots, K\}$, $k \neq s$. This is stronger than assuming the margin condition only for the difference between the largest and the second-to-largest $\theta_k(\mathbf{X})$. In this light, depending on the form of g_k and f_k, better bounds under weaker conditions might be obtained.

S2 Proofs of Results in Section 2

S2.1 Proof of Theorem 1

The argument of Balke and Pearl (1997) is valid within levels of $\boldsymbol{X}$, given Assumptions 1–4. This implies that for each $\boldsymbol{x} \in \mathcal{X}$,

$$\gamma_\ell(\boldsymbol{x}) \leq \mathbb{E}(Y(a=1) - Y(a=0) \mid \boldsymbol{X} = \boldsymbol{x}) \leq \gamma_u(\boldsymbol{x}), \tag{2}$$

and that these bounds are tight. It follows that for any $\epsilon > 0$, there exist joint distributions $\mathbb{P}^*_{1,\epsilon}, \mathbb{P}^*_{2,\epsilon}$ on the full data $(\boldsymbol{X}, Z, A, Y(a=0), Y(a=1))$, compatible with the observed data law $\mathbb{P}$ such that

$$\mathbb{P}\left(\mathbb{E}_{\mathbb{P}^*_{1,\epsilon}}(Y(a=1) - Y(a=0) \mid \boldsymbol{X}) < \gamma_\ell(\boldsymbol{X}) + \epsilon\right) = 1, \tag{3}$$

and

$$\mathbb{P}\left(\mathbb{E}_{\mathbb{P}^*_{2,\epsilon}}(Y(a=1) - Y(a=0) \mid \boldsymbol{X}) > \gamma_u(\boldsymbol{X}) - \epsilon\right) = 1.$$

By iterated expectations, $\mathbb{E}_{\mathbb{P}}(\gamma_\ell(\boldsymbol{X}))$ and $\mathbb{E}_{\mathbb{P}}(\gamma_u(\boldsymbol{X}))$ represent valid lower and upper bounds, respectively, on the ATE. To prove that these are also tight, we proceed by contradiction: we focus on the lower bound without loss of generality, and assume there exists $\epsilon > 0$ such that for all full data laws $\mathbb{P}^*$ compatible with $\mathbb{P}$, $\mathbb{E}_{\mathbb{P}^*}(Y(a=1) - Y(a=0)) \geq \mathbb{E}_{\mathbb{P}}(\gamma_\ell(\boldsymbol{X})) + \epsilon$. But (3) implies $\mathbb{E}_{\mathbb{P}^*_{1,\epsilon}}(Y(a=1) - Y(a=0)) < \mathbb{E}_{\mathbb{P}}(\gamma_\ell(\boldsymbol{X})) + \epsilon$, which yields a contradiction.

S2.2 Proof of Proposition 1

The bounds based on $(\boldsymbol{X}, \boldsymbol{G})$ are of the same form as those for $\boldsymbol{X}$ alone, but with underlying probabilities $\pi^\dagger_{ya.z}(\boldsymbol{X}, \boldsymbol{G}) = \mathbb{P}[Y = y, A = a \mid \boldsymbol{X}, \boldsymbol{G}, Z = z]$ instead of $\pi_{ya.z}(\boldsymbol{X})$. The key observation is that for any $y, a, z \in \{0, 1\}$,

$$\pi_{ya.z}(\boldsymbol{X}) = \mathbb{E}(\pi^\dagger_{ya.z}(\boldsymbol{X}, \boldsymbol{G}) \mid \boldsymbol{X}, Z = z) = \mathbb{E}(\pi^\dagger_{ya.z}(\boldsymbol{X}, \boldsymbol{G}) \mid \boldsymbol{X}),$$

where the first equality holds by the tower law, and the second holds by the assumption $Z \perp\!\!\!\perp \boldsymbol{G} \mid \boldsymbol{X}$. Therefore, letting $\theta^\dagger_{\ell,j}(\boldsymbol{X}, \boldsymbol{G})$, $\theta^\dagger_{u,j}(\boldsymbol{X}, \boldsymbol{G})$ be of the same for as $\theta_{\ell,j}(\boldsymbol{X})$, $\theta_{u,j}(\boldsymbol{X})$, respectively, for $j = 1, \ldots, 8$, but with $\pi^\dagger_{ya.z}$ replacing $\pi_{ya.z}$ for all $y, a, z \in \{0, 1\}$, we have

$$\mathbb{E}_{\mathbb{P}}\left(\max_{1 \leq j \leq 8} \theta_{\ell,j}(\boldsymbol{X})\right) = \mathbb{E}_{\mathbb{P}}\left(\max_{1 \leq j \leq 8} \mathbb{E}_{\mathbb{P}}(\theta^\dagger_{\ell,j}(\boldsymbol{X}, \boldsymbol{G}) \mid \boldsymbol{X})\right) \leq \mathbb{E}_{\mathbb{P}}\left(\max_{1 \leq j \leq 8} \theta^\dagger_{\ell,j}(\boldsymbol{X}, \boldsymbol{G})\right),$$

where we used a conditional version of Jensen's inequality for the pointwise maximum, and iterated expectations. The proof that the $(\boldsymbol{X}, \boldsymbol{G})$-upper bound is lower follows by the same logic, using concavity of the pointwise minimum. The example in Section 3.2 shows that the lower (upper) bound based on $(\boldsymbol{X}, \boldsymbol{G})$ may be strictly greater (smaller).

S2.3 Proof of Corollary 1

This follows immediately by Proposition 1, replacing $\boldsymbol{X}$ with $\emptyset$, and $\boldsymbol{G}$ with $\boldsymbol{X}$.

S2.4 Simplification under Monotonicity

The bounds $\gamma_\ell(\boldsymbol{X}) = \max_{1\le j\le 8} \theta_{\ell,j}(\boldsymbol{X})$ and $\gamma_u(\boldsymbol{X}) = \min_{1\le j\le 8} \theta_{u,j}(\boldsymbol{X})$ on the CATE due to Balke and Pearl (1997) are often compared to simpler valid bounds derived earlier in Robins (1989); Manski (1990): in our setting with baseline confounders, $\beta_\ell(\boldsymbol{X}) \le \mathbb{E}(Y(1) - Y(0) \mid \boldsymbol{X}) \le \beta_u(\boldsymbol{X})$, where

$$\beta_\ell(\boldsymbol{X}) = \mathbb{E}_P(AY \mid \boldsymbol{X}, Z = 1) - \mathbb{E}_P(Y(1-A) + A \mid \boldsymbol{X}, Z = 0),$$

and

$$\beta_u(\boldsymbol{X}) = \mathbb{E}_P(AY + 1 - A \mid \boldsymbol{X}, Z = 1) - \mathbb{E}_P(Y(1-A) \mid \boldsymbol{X}, Z = 0).$$

Balke and Pearl (1997) show that their bounds on the treatment effect are in general tighter than these simpler bounds, as seen in the following result.

Lemma 1. $\beta_\ell(\boldsymbol{X}) = \theta_{\ell,1}(\boldsymbol{X}) \le \gamma_\ell(\boldsymbol{X}) \le \gamma_u(\boldsymbol{X}) \le \theta_{u,1}(\boldsymbol{X}) = \beta_u(\boldsymbol{X})$.

Proof. Observe that, noting $A = YA + (1-Y)A$,

$$\begin{aligned}\beta_\ell(\boldsymbol{X}) &= \mathbb{E}_P(AY \mid \boldsymbol{X}, Z = 1) - \mathbb{E}_P(Y(1-A) + A \mid \boldsymbol{X}, Z = 0)\\ &= \pi_{11.1}(\boldsymbol{X}) - \pi_{10.0}(\boldsymbol{X}) - \pi_{11.0}(\boldsymbol{X}) - \pi_{01.0}(\boldsymbol{X})\\ &= \pi_{11.1}(\boldsymbol{X}) - \{1 - \pi_{00.0}(\boldsymbol{X})\}\\ &= \theta_{\ell,1}(\boldsymbol{X}).\end{aligned}$$

Similarly,

$$\begin{aligned}\beta_u(\boldsymbol{X}) &= \mathbb{E}_P(AY + 1 - A \mid \boldsymbol{X}, Z = 1) - \mathbb{E}_P(Y(1-A) \mid \boldsymbol{X}, Z = 0)\\ &= \pi_{11.1}(\boldsymbol{X}) + \pi_{10.1}(\boldsymbol{X}) + \pi_{00.1}(\boldsymbol{X}) - \pi_{10.0}(\boldsymbol{X})\\ &= \{1 - \pi_{01.1}(\boldsymbol{X})\} - \pi_{10.0}(\boldsymbol{X})\\ &= \theta_{u,1}(\boldsymbol{X}).\end{aligned}$$

□

These simpler bounds are known to be tight — see Theorem 7.3 of Balke and Pearl (1993) — when we additionally assert the monotonicity assumption, i.e., $A(z = 1) \ge A(z = 0)$ almost surely. Thus, somewhat surprisingly, monotonicity does not enable us to obtain tighter bounds, but does simplify the structure in that $\beta_\ell(\boldsymbol{X}) = \gamma_\ell(\boldsymbol{X})$ and $\beta_u(\boldsymbol{X}) = \gamma_u(\boldsymbol{X})$. A consequence of this analysis is that in the special case of a randomized trial with monotonicity, covariate-assisted bounds offer no reduction in width for bounding the marginal ATE compared to covariate-agnostic bounds

$$\beta_\ell^*(P) = \mathbb{E}_P(AY \mid Z = 1) - \mathbb{E}_P(Y(1-A) + A \mid Z = 0),$$

and

$$\beta_u^*(P) = \mathbb{E}_P(AY + 1 - A \mid Z = 1) - \mathbb{E}_P(Y(1-A) \mid Z = 0),$$

since $\beta_\ell^*(P) = \mathbb{E}_P(\beta_\ell(\boldsymbol{X}))$ and $\beta_u^*(P) = \mathbb{E}_P(\beta_u(\boldsymbol{X}))$ by randomization, i.e., $Z \perp\!\!\!\perp \boldsymbol{X}$. That said, one upside is that we can leverage randomization to obtain more efficient estimators of the bounds in this setting compared to nonparametric estimators of $\beta_\ell^*(P)$ and $\beta_u^*(P)$. Indeed, the efficient influence functions of the bounds in this setting will be the nonparametric influence functions of the functionals $\mathbb{E}_P(\beta_\ell(\boldsymbol{X}))$ and $\mathbb{E}_P(\beta_u(\boldsymbol{X}))$—we omit this analysis here.

S3 Proofs of Results in Section 4

S3.1 Proof of Theorem 2

The proof structures follows that of the proof of Theorem 4, explained in greater detail below. We prove the result for the lower bound as the result for the upper bound is analogous. By the standard decomposition, we have

$$\begin{aligned}\widehat{\mathcal{L}} - \mathcal{L} &= (\mathbb{P}_n - \mathbb{P})\{\varphi_\ell(O;\widehat{\mathbb{P}},\widehat{d}_\ell) - \varphi_\ell(O;\mathbb{P},d_\ell)\} + \mathbb{P}\{\varphi_\ell(O;\widehat{\mathbb{P}},\widehat{d}_\ell) - \varphi_\ell(O;\mathbb{P},d_\ell)\} \\ &\quad + (\mathbb{P}_n - \mathbb{P})\{\varphi_\ell(O;\mathbb{P},d_\ell)\} \\ &\equiv R_1 + R_2 + (\mathbb{P}_n - \mathbb{P})\{\varphi_\ell(O;\mathbb{P},d_\ell)\}\end{aligned}$$

We will show that $R_1 = o_{\mathbb{P}}(n^{-1/2})$ and

$$R_2 = O_{\mathbb{P}}\left(\left\|\widehat{\lambda}_1 - \lambda_1\right\| \cdot \max_{y,a,z\in\{0,1\}} \|\widehat{\pi}_{ya.z} - \pi_{ya.z}\| + \max_{1\le j\le 8}\left\|\widehat{\theta}_{\ell,j} - \theta_{\ell,j}\right\|_\infty^{1+\alpha}\right)$$

under the conditions of the theorem.

S3.1.1 Term R_1

By Lemma 2 in Kennedy et al. (2020), $R_1 = o_{\mathbb{P}}(n^{-1/2})$ if

$$\int \{\varphi_\ell(o;\widehat{\mathbb{P}},\widehat{d}_\ell) - \varphi_\ell(o;\mathbb{P},d_\ell)\}^2 d\mathbb{P}(o) = o_{\mathbb{P}}(1)$$

We have

$$\begin{aligned}\int \{\varphi_\ell(o;\widehat{\mathbb{P}},\widehat{d}_\ell) - \varphi_\ell(o;\mathbb{P},d_\ell)\}^2 d\mathbb{P}(o) &\lesssim \int \{\varphi_\ell(o;\widehat{\mathbb{P}},\widehat{d}_\ell) - \varphi_\ell(o;\mathbb{P},\widehat{d}_\ell)\}^2 d\mathbb{P}(o) \\ &\quad + \int \{\varphi_\ell(o;\mathbb{P},\widehat{d}_\ell) - \varphi_\ell(o;\mathbb{P},d_\ell)\}^2 d\mathbb{P}(o)\end{aligned}$$

For the first term,

$$\int \{\varphi_\ell(o;\widehat{\mathbb{P}},\widehat{d}_\ell) - \varphi_\ell(o;\mathbb{P},\widehat{d}_\ell)\}^2 d\mathbb{P}(o) \lesssim \sum_{j=1}^{8} \int \{L_j(o;\widehat{\mathbb{P}}) - L_j(o;\mathbb{P}) + \widehat{\theta}_{\ell,j} - \theta_{\ell,j}\}^2 d\mathbb{P}(o) = o_{\mathbb{P}}(1)$$

since for example for $j=1$, we have

$$\begin{aligned}&\left\|(\widehat{\pi}_{11.1} - \pi_{11.1})\left(1 - \frac{\mathbb{1}(Z=1)}{\widehat{\lambda}_1}\right) + \frac{\mathbb{1}(Z=1)}{\widehat{\lambda}_1\lambda_1}\{\mathbb{1}(Y=1,A=1) - \pi_{11.1}\}\left(\lambda_1 - \widehat{\lambda}_1\right)\right\| \\ &\quad + \left\|(\widehat{\pi}_{00.0} - \pi_{00.0})\left(1 - \frac{\mathbb{1}(Z=0)}{\widehat{\lambda}_0}\right) + \frac{\mathbb{1}(Z=0)}{\widehat{\lambda}_0\lambda_0}\{\mathbb{1}(Y=0,A=0) - \pi_{00.0}\}\left(\lambda_0 - \widehat{\lambda}_0\right)\right\| \\ &\lesssim \left\|\widehat{\lambda}_1 - \lambda_1\right\| + \max_{y,a,z\in\{0,1\}} \|\widehat{\pi}_{ya.z} - \pi_{ya.z}\| = o_P(1),\end{aligned}$$

by our assumptions, using the fact that $\widehat{\lambda}_z$ and λ_z are bounded away from zero.

For the second term, we have

$$\begin{aligned}\int \{\varphi_\ell(o;\mathbb{P},\widehat{d}_\ell) - \varphi_\ell(o;\mathbb{P},d_\ell)\}^2 d\mathbb{P}(o) &= \sum_{j=1}^{8} \int \left|\mathbb{1}\{\widehat{d}_\ell(\mathbf{x}) = j\} - \mathbb{1}\{d_\ell(\mathbf{x}) = j\}\right| \{L_j(o;\mathbb{P}) + \theta_{\ell,j}(\mathbf{x})\}^2 d\mathbb{P}(o) \\ &\lesssim \mathbb{P}\left\{\theta_{\ell,\widehat{d}(\boldsymbol{X})}(\boldsymbol{X}) \neq \theta_{\ell,d_\ell(\boldsymbol{X})}(\boldsymbol{X})\right\}\end{aligned}$$

since $\theta_{\ell,j}(\boldsymbol{X})$ and $L_j(O;\mathbb{P})$ are all uniformly bounded. Next, we show that

$$\mathbb{P}\left\{\theta_{\ell,\widehat{d}(\boldsymbol{X})}(\boldsymbol{X}) \neq \theta_{\ell,d_\ell(\boldsymbol{X})}(\boldsymbol{X})\right\} = o_{\mathbb{P}}(1)$$

For any $t > 0$, we have

$$\begin{aligned}
\mathbb{P}\left[\theta_{\ell,\widehat{d}_\ell(\boldsymbol{X})} \neq \theta_{\ell,d_\ell(\boldsymbol{X})}\right] &= \mathbb{P}\left[\theta_{\ell,\widehat{d}_\ell(\boldsymbol{X})} \neq \theta_{\ell,d_\ell(\boldsymbol{X})}, \min_{j\neq d_\ell(\boldsymbol{X})}\{\theta_{\ell,d_\ell(\boldsymbol{X})}(\boldsymbol{X}) - \theta_{\ell,j}(\boldsymbol{X})\} \leq t\right] \\
&\quad + \mathbb{P}\left[\theta_{\ell,\widehat{d}_\ell(\boldsymbol{X})} \neq \theta_{\ell,d_\ell(\boldsymbol{X})}, \min_{j\neq d_\ell(\boldsymbol{X})}\{\theta_{\ell,d_\ell(\boldsymbol{X})}(\boldsymbol{X}) - \theta_{\ell,j}(\boldsymbol{X})\} > t\right] \\
&\leq \mathbb{P}\left[\min_{j\neq d_\ell(\boldsymbol{X})}\{\theta_{\ell,d_\ell(\boldsymbol{X})}(\boldsymbol{X}) - \theta_{\ell,j}(\boldsymbol{X})\} \leq t\right] + \mathbb{P}\left[\theta_{\ell,d_\ell(\boldsymbol{X})} - \theta_{\ell,\widehat{d}_\ell(\boldsymbol{X})} > t\right] \\
&\leq Ct^\alpha + \mathbb{P}\left[\theta_{\ell,d_\ell(\boldsymbol{X})} - \theta_{\ell,\widehat{d}_\ell(\boldsymbol{X})} + \widehat{\theta}_{\ell,\widehat{d}_\ell(\boldsymbol{X})} - \widehat{\theta}_{\ell,d_\ell(\boldsymbol{X})} > t\right] \\
&\leq Ct^\alpha + \mathbb{P}\left\{2\sum_{j=1}^{8}|\widehat{\theta}_{\ell,j} - \theta_{\ell,j}| > t\right\} \\
&\leq Ct^\alpha + \frac{2}{t}\sum_{j=1}^{8}\mathbb{P}|\widehat{\theta}_{\ell,j}(\boldsymbol{X}) - \theta_{\ell,j}(\boldsymbol{X})| \\
&\leq Ct^\alpha + \frac{2}{t}\sum_{j=1}^{8}\left\|\widehat{\theta}_{\ell,j} - \theta_{\ell,j}\right\|
\end{aligned}$$

where $C > 0$ is the universal constant in Assumption 5. In the second line, we use that $\theta_{\ell,\widehat{d}_\ell(\boldsymbol{X})} \neq \theta_{\ell,d_\ell(\boldsymbol{X})}$ implies $\widehat{d}_\ell(\boldsymbol{X}) \neq d_\ell(\boldsymbol{X})$, so $\theta_{\ell,d_\ell(\boldsymbol{X})} - \theta_{\ell,\widehat{d}_\ell(\boldsymbol{X})} \geq \min_{j\neq d_\ell(\boldsymbol{X})}\{\theta_{\ell,d_\ell(\boldsymbol{X})}(\boldsymbol{X}) - \theta_{\ell,j}(\boldsymbol{X})\}$. In the third line we use Assumption 5 and that $\widehat{\theta}_{\ell,\widehat{d}_\ell(\boldsymbol{X})}(\boldsymbol{X}) - \widehat{\theta}_{\ell,d_\ell(\boldsymbol{X})}(\boldsymbol{X}) \geq 0$ by construction of $\widehat{d}_\ell(\boldsymbol{X})$. The fourth line follows from Markov's inequality, and the last line from $\|\cdot\|_{L_1} \leq \|\cdot\|_{L_2}$. Since for each $j \in \{1,\ldots,8\}$ we have $\left\|\widehat{\theta}_{\ell,j} - \theta_{\ell,j}\right\| = o_P(1)$, as each $\widehat{\theta}_{\ell,j} - \theta_{\ell,j}$ is a linear combination of the differences $\{\widehat{\pi}_{ya.z} - \pi_{ya.z} : y,a,z \in \{0,1\}\}$, we obtain the desired result by invoking Lemma 2. Namely, we set $X_n = \mathbb{P}\left[\theta_{\ell,\widehat{d}_\ell(\boldsymbol{X})} \neq \theta_{\ell,d_\ell(\boldsymbol{X})}\right]$, and for any $\epsilon > 0$, choose $t_\epsilon = \left(\frac{\epsilon}{C}\right)^{1/\alpha} > 0$ and $Z_n^{(\epsilon)} = \frac{2}{t_\epsilon}\sum_{j=1}^{8}\left\|\widehat{\theta}_{\ell,j} - \theta_{\ell,j}\right\|$.

Lemma 2. *Suppose that for a given sequence X_n, one can find for any $\epsilon > 0$ another sequence $Z_n^{(\epsilon)} \geq 0$ such that $|X_n| \leq \epsilon + Z_n^{(\epsilon)}$ and $Z_n^{(\epsilon)} = o_P(1)$. Then $X_n = o_P(1)$.*

Proof. Fixing $\epsilon > 0$, consider a non-negative sequence $Z_n^{(\epsilon/2)} = o_P(1)$ satisfying $|X_n| \leq \epsilon/2 + Z_n^{(\epsilon/2)}$. Then

$$P[|X_n| > \epsilon] \leq P[\epsilon/2 + Z_n^{(\epsilon/2)} > \epsilon] = P[Z_n^{(\epsilon/2)} > \epsilon/2] \to 0 \text{ as } n \to \infty,$$

since $Z_n = o_P(1)$, thus proving the result. □

S3.1.2 Term R_2

We decompose R_2 as

$$R_2 = \sum_{j=1}^{8} \Big\{ \mathbb{P}\left[\mathbb{1}\{\widehat{d}_\ell(\boldsymbol{X}) = j\}\{L_j(O;\widehat{\mathbb{P}}) + \widehat{\theta}_{\ell,j}(\boldsymbol{X}) - \theta_{\ell,j}(\boldsymbol{X})\}\right] \\ + \mathbb{P}\left(\left[\mathbb{1}\{\widehat{d}_\ell(\boldsymbol{X}) = j\} - \mathbb{1}\{d_\ell(\boldsymbol{X}) = j\}\right] \theta_{\ell,j}(\boldsymbol{X}) \right) \Big\}$$

We have

$$\mathbb{P}\left[\mathbb{1}\{\widehat{d}_\ell(\boldsymbol{X}) = j\}\{L_j(O;\widehat{\mathbb{P}}) + \widehat{\theta}_{\ell,j}(\boldsymbol{X}) - \theta_{\ell,j}(\boldsymbol{X})\}\right] \lesssim \left\| \widehat{\lambda}_1 - \lambda_1 \right\| \cdot \max_{y,a,z \in \{0,1\}} \|\widehat{\pi}_{ya.z} - \pi_{ya.z}\|$$

For the second term, observe that

$$\begin{aligned}
&\left| \mathbb{P}\left\{ \theta_{\ell,\widehat{d}_\ell(\boldsymbol{X})}(\boldsymbol{X}) - \theta_{\ell,d_\ell(\boldsymbol{X})}(\boldsymbol{X}) \right\} \right| \\
&= \left| \mathbb{P}\left[\mathbb{1}\{\theta_{\ell,d_\ell(\boldsymbol{X})}(\boldsymbol{X}) > \theta_{\ell,\widehat{d}_\ell(\boldsymbol{X})}(\boldsymbol{X})\} \left\{ \theta_{\ell,d_\ell(\boldsymbol{X})}(\boldsymbol{X}) - \theta_{\ell,\widehat{d}_\ell(\boldsymbol{X})}(\boldsymbol{X}) \right\} \right] \right| \\
&\leq \mathbb{P}\Bigg(\mathbb{1}\left(\min_{j \neq d_\ell(\boldsymbol{X})} \{\theta_{\ell,d_\ell(\boldsymbol{X})} - \theta_{\ell,j}\} \leq \theta_{\ell,d_\ell(\boldsymbol{X})} - \theta_{\ell,\widehat{d}_\ell(\boldsymbol{X})} + \widehat{\theta}_{\ell,\widehat{d}_\ell(\boldsymbol{X})} - \widehat{\theta}_{\ell,d_\ell(\boldsymbol{X})} \right) \\
&\qquad \times \left(\theta_{\ell,d_\ell(\boldsymbol{X})} - \theta_{\ell,\widehat{d}_\ell(\boldsymbol{X})} + \widehat{\theta}_{\ell,\widehat{d}_\ell(\boldsymbol{X})} - \widehat{\theta}_{\ell,d_\ell(\boldsymbol{X})} \right) \Bigg) \\
&\leq 2 \max_{1 \leq j \leq 8} \left\| \widehat{\theta}_{\ell,j} - \theta_{\ell,j} \right\|_\infty \mathbb{P}\left[\min_{j \neq d_\ell(\boldsymbol{X})} \{\theta_{\ell,d_\ell(\boldsymbol{X})} - \theta_{\ell,j}\} \leq 2 \max_{1 \leq j \leq 8} \left\| \widehat{\theta}_{\ell,j} - \theta_{\ell,j} \right\|_\infty \right] \\
&\lesssim \max_{1 \leq j \leq 8} \left\| \widehat{\theta}_{\ell,j} - \theta_{\ell,j} \right\|_\infty^{1+\alpha},
\end{aligned}$$

by Assumption 5, where we used the fact that $\theta_{\ell,d_\ell(\boldsymbol{X})}(\boldsymbol{X}) \geq \theta_{\ell,\widehat{d}_\ell(\boldsymbol{X})}(\boldsymbol{X})$ and $\widehat{\theta}_{\ell,d_\ell(\boldsymbol{X})}(\boldsymbol{X}) \leq \widehat{\theta}_{\ell,\widehat{d}_\ell(\boldsymbol{X})}(\boldsymbol{X})$, by construction of d_ℓ and $\widehat{d}_\ell$.

S4 Elaboration and Proofs of Results in Section 5

S4.1 Doubly Robust Machine Learning Framework

We now briefly review the statistical framework we use to derive our estimators and to evaluate the theoretical properties of our methods. A central goal is to develop methods that are flexible and resistant to bias from model misspecification.

Here, we reduce the potential for model misspecification by using flexible nonparametric machine learning (ML) tools. More specifically, we aim to construct bias-corrected estimators using influence functions—a central element of semiparametric theory. As an example, we review estimation of the mean counterfactual, $\mathbb{E}(Y(a=1))$ (i.e., the mean outcome if every unit in the population were treated), from this perspective. For the purposes of illustration, we assume (just for this paragraph) that the data consist of n iid copies of $O = (\boldsymbol{X}, A, Y) \sim \mathbb{P}$. Under no unmeasured confounding and other assumptions, $\mathbb{E}(Y(a=1))$ can be written as an averaged regression function (Robins, 1986): $\psi(\mathbb{P}) = \mathbb{E}_{\mathbb{P}}\{\mathbb{E}_{\mathbb{P}}(Y \mid A = 1, \boldsymbol{X})\}$. Estimating $\mathbb{E}_{\mathbb{P}}(Y \mid A = 1, \boldsymbol{X})$ is a standard regression problem and flexible ML methods may be preferred to more restrictive parametric estimation methods such as linear regression to avoid model misspecification and reduce bias. However, if ψ is estimated as the average of predictions from

a ML model, rather than from a parametric model, the estimate will generally inherit first-order smoothing bias from the nonparametric estimates. Instead, one can find a function of the data, which we can denote generically as φ, such that estimating ψ as the average value of (an estimated version of) φ will correct this first-order bias. An optimal choice of this function is referred to as the *influence function* of ψ, and in this case is based on two regression functions, $\mathbb{E}_{\mathbb{P}}(Y \mid A = 1, \boldsymbol{X})$ and $\mathbb{P}(A = 1 \mid \boldsymbol{X})$. In practice, the analyst fits two models: a model for the outcome regressed against the treatment and all confounders (the outcome model); and a model regressing the treatment against all confounders (the propensity model). These models are combined to estimate the effect of interest. This approach is "doubly robust", since it is consistent if either the propensity model or the outcome model is correctly specified (Scharfstein et al., 1999), and also leads to "doubly reduced" second-order bias. Finally, the estimation process is combined with sample-splitting or cross-fitting, to prevent over-fitting (or formally, to avoid imposing complexity restrictions on the class of nuisance estimators) by separating estimation of the components of the influence function from estimation of its mean (Robins et al., 2008; Zheng and van der Laan, 2010; Chernozhukov et al., 2018).

In semiparametric efficiency theory (Bickel et al., 1993; Tsiatis, 2006; van der Vaart, 2000; Kennedy, 2016), a fundamental goal is to characterize the (efficient) influence function. Mathematically, an influence function is the derivative in a von Mises expansion of the target statistical functional (analogous to the usual derivative of a function in Taylor expansion). In robust statistics, it coincides with the Gateaux derivative of the functional in the direction of a point-mass contamination distribution. The influence function serves a number of purposes. First, the variance of the efficient influence function is equal to the efficiency bound of the target statistical functional, which serves as a lower bound of the variance for regular estimators. It characterizes the inherent estimation difficulty of the target functional and provides a benchmark to compare against when we construct estimators. Moreover, it enables us to correct for first-order bias in the plug-in estimator and motivates the robust estimator, which has a general second-order bias property so that nonparametric and flexible machine learning methods with relatively slow rates can be used for estimating the nuisance functions.

S4.2 Background on log-sum-exp Function

The log-sum-exp (LSE) function is commonly employed across a range of disciplines, including statistical mechanics (Aldous, 2005) and machine learning (Calafiore et al., 2020). Using the LSE function to approximate the maximum function is also common in the statistics literature. For instance, one way to prove Sudakov-Fernique's inequality on Gaussian comparison is to apply the functional form of Slepian's inequality (which requires the function considered to be twice-differentiable) to the LSE function and let $t \to \infty$ (Vershynin, 2018; Wainwright, 2019). As another example, in proving a high-dimensional Gaussian comparison version of the central limit theorem, Chernozhukov et al. (2012) used Slepian's Gaussian interpolation together with Stein's leave-one-out expansions. A typical tool to evaluate Stein's leave-one-out expansion is a Taylor's expansion, which requires differentiability of the function. Hence, Chernozhukov et al. (2012) also approximated the maximum function with the LSE function, and selected the tuning parameter t to limit approximation error while controlling the derivatives of the LSE function.

S4.3 Proof of Theorem 3

Recall an influence function of a pathwise differentiable functional $\chi(\mathbb{P})$, at $\mathbb{P}$ in a given statistical model, is a zero-mean finite-variance function $\dot{\chi}(O;\mathbb{P})$ of observed data O such that for any regular one-dimensional parametric submodel $\mathbb{P}_\epsilon$ through $\mathbb{P}_0 = \mathbb{P}$, it holds that

$$\frac{d}{d\epsilon}\chi\left(\mathbb{P}_\epsilon\right)\bigg|_{\epsilon=0} = \mathbb{E}_{\mathbb{P}}[\dot{\chi}(O;\mathbb{P})u(O)]$$

where $u(O)$ is the score function of the parametric submodel at $\mathbb{P}$. For such a parametric submodel, invoking the total derivative, we have (let $\boldsymbol{\theta}_{\epsilon,\ell}(\boldsymbol{X})$ be the nuisance function vector at submodel $\mathbb{P}_\epsilon$)

$$\begin{aligned}
&\frac{d}{d\epsilon}\mathcal{L}_g\left(\mathbb{P}_\epsilon\right)\bigg|_{\epsilon=0} \\
=\ &\frac{d}{d\epsilon}\mathbb{E}_{\mathbb{P}_\epsilon}\left[g\left(\boldsymbol{\theta}_{\epsilon,\ell}(\boldsymbol{X})\right)\right]\bigg|_{\epsilon=0} \\
=\ &\frac{d}{d\epsilon}\mathbb{E}_{\mathbb{P}_\epsilon}\left[g\left(\boldsymbol{\theta}_{\ell}(\boldsymbol{X})\right)\right]\bigg|_{\epsilon=0} + \sum_{j=1}^{8}\frac{d}{d\epsilon}\mathbb{E}_{\mathbb{P}}\left[g\left(\theta_{\ell,1}(\boldsymbol{X}),\dots,\theta_{\epsilon,\ell,j}(\boldsymbol{X}),\dots,\theta_{\ell,8}(\boldsymbol{X})\right)\right]\bigg|_{\epsilon=0} \\
=\ &\mathbb{E}_{\mathbb{P}}\left[\left(g\left(\boldsymbol{\theta}_{\ell}(\boldsymbol{X})\right)-\mathcal{L}_g(\mathbb{P})\right)u(O)\right] + \sum_{j=1}^{8}\mathbb{E}_{\mathbb{P}}\left[\frac{\partial g\left(\boldsymbol{\theta}_{\ell}(\boldsymbol{X})\right)}{\partial\theta_{\ell,j}(\boldsymbol{X})}\frac{d}{d\epsilon}\theta_{\epsilon,\ell,j}(\boldsymbol{X})\bigg|_{\epsilon=0}\right]
\end{aligned} \tag{4}$$

using the fact that score function u has mean zero to center the first term, and the chain rule for the remaining eight terms. Next, observe that, for any $(y,a,z)\in\{0,1\}^3$, (denote in general $u(B \mid C)$ as the conditional score function for the distribution of B given C.

$$\begin{aligned}
\frac{d}{d\epsilon}\pi_{\epsilon,ya.z}(\boldsymbol{X})\bigg|_{\epsilon=0} &= \mathbb{E}_{\mathbb{P}}\left[\left(\mathbb{1}(Y=y,A=a)-\pi_{ya.z}(\boldsymbol{X})\right)u(Y,A\mid Z=z,\boldsymbol{X})\mid Z=z,\boldsymbol{X}\right] \\
&= \mathbb{E}_{\mathbb{P}}\left[\frac{\mathbb{1}(Z=z)}{\lambda_z(\boldsymbol{X})}\left\{\mathbb{1}(Y=y,A=a)-\pi_{ya.z}(\boldsymbol{X})\right\}u(Y,A\mid Z=z,\boldsymbol{X})\mid\boldsymbol{X}\right] \\
&= \mathbb{E}_{\mathbb{P}}\left[\frac{\mathbb{1}(Z=z)}{\lambda_z(\boldsymbol{X})}\left\{\mathbb{1}(Y=y,A=a)-\pi_{ya.z}(\boldsymbol{X})\right\}u(Y,A\mid Z,\boldsymbol{X})\mid\boldsymbol{X}\right] \\
&= \mathbb{E}_{\mathbb{P}}\left(\psi_{ya.z}(O;\mathbb{P})u(Y,A\mid Z,\boldsymbol{X})\mid\boldsymbol{X}\right),
\end{aligned} \tag{5}$$

where the first equation follows from directly taking derivatives and centering with $\pi_{ya.z}$ since $u(Y,A \mid Z=z,\boldsymbol{X})$ has conditional mean zero. The second equation follows from conditioning on $Z,\boldsymbol{X}$ and using property of conditional expectations. The last equation follows from the definition of $\psi_{ya.z}$.

Since $\theta_{\ell,j}$'s are linear combinations of $\pi_{ya.z}$ (and constant 1), we can prove

$$\mathbb{E}_{\mathbb{P}}\left[\frac{\partial g\left(\boldsymbol{\theta}_{\ell}(\boldsymbol{X})\right)}{\partial\theta_{\ell,j}(\boldsymbol{X})}\frac{d}{d\epsilon}\theta_{\epsilon,\ell,j}(\boldsymbol{X})\bigg|_{\epsilon=0}\right] = \mathbb{E}_{\mathbb{P}}\left[\frac{\partial g\left(\boldsymbol{\theta}_{\ell}(\boldsymbol{X})\right)}{\partial\theta_{\ell,j}(\boldsymbol{X})}L_j(O;\mathbb{P})u(O)\right].$$

For illustration, take $j = 1$ (other j's can be proved similarly) and we have

$$
\begin{aligned}
&\mathbb{E}_{\mathbb{P}}\left[\frac{\partial g\left(\boldsymbol{\theta}_{\ell}(\boldsymbol{X})\right)}{\partial \theta_{\ell,1}(\boldsymbol{X})} \frac{d}{d\epsilon}\theta_{\epsilon,\ell,1}(\boldsymbol{X})\Big|_{\epsilon=0}\right] \\
=&\mathbb{E}_{\mathbb{P}}\left[\frac{\partial g\left(\boldsymbol{\theta}_{\ell}(\boldsymbol{X})\right)}{\partial \theta_{\ell,1}(\boldsymbol{X})} \frac{d}{d\epsilon}\left\{\pi_{\epsilon,11.1}(\boldsymbol{X}) + \pi_{\epsilon,00.0}(\boldsymbol{X}) - 1\right\}\Big|_{\epsilon=0}\right] \\
=&\mathbb{E}_{\mathbb{P}}\left[\frac{\partial g\left(\boldsymbol{\theta}_{\ell}(\boldsymbol{X})\right)}{\partial \theta_{\ell,1}(\boldsymbol{X})} \mathbb{E}_{\mathbb{P}}\left[\left\{\psi_{11.1}(O;\mathbb{P}) + \psi_{00.0}(O;\mathbb{P})\right\} u(Y, A \mid Z, \boldsymbol{X}) \mid \boldsymbol{X}\right]\right] \\
=&\mathbb{E}_{\mathbb{P}}\left[\frac{\partial g\left(\boldsymbol{\theta}_{\ell}(\boldsymbol{X})\right)}{\partial \theta_{\ell,1}(\boldsymbol{X})} \left\{\psi_{11.1}(O;\mathbb{P}) + \psi_{00.0}(O;\mathbb{P})\right\} u(Y, A \mid Z, \boldsymbol{X})\right] \\
=&\mathbb{E}_{\mathbb{P}}\left[\frac{\partial g\left(\boldsymbol{\theta}_{\ell}(\boldsymbol{X})\right)}{\partial \theta_{\ell,1}(\boldsymbol{X})} L_1(O;\mathbb{P}) u(O)\right],
\end{aligned}
$$

where the first equation follows from definition of $\theta_{\epsilon,\ell,1}$. The second equation follows from (5). The third equation follows from property of conditional distribution. In the final equation we added $u(Z, \boldsymbol{X})$ and noted $u(O) = u(Z, \boldsymbol{X}) + u(Y, A \mid Z, \boldsymbol{X})$ — we are permitted to add this term as it is a function only of (Z, X), and $\psi_{ya.z}(O;\mathbb{P})$ has mean zero given $(Z, \boldsymbol{X})$.

Plugging these equations into (4) we have

$$
\frac{d}{d\epsilon}\mathcal{L}_g\left(\mathbb{P}_{\epsilon}\right)\Big|_{\epsilon=0} = \mathbb{E}_{\mathbb{P}}\left\{\left[g\left(\boldsymbol{\theta}_{\ell}(\boldsymbol{X})\right) - \mathcal{L}_g(\mathbb{P}) + \sum_{j=1}^{8} \frac{\partial g\left(\boldsymbol{\theta}_{\ell}(\boldsymbol{X})\right)}{\partial \theta_{\ell,j}(\boldsymbol{X})} L_j(O;\mathbb{P})\right] u(O)\right\}.
$$

The argument for deriving the influence function of $\mathcal{U}_h(\mathbb{P})$ uses the exact same logic.

S4.4 Proof of Theorem 4

We first prove a proposition that characterizes the conditional bias of the robust estimator. In the following derivations all the expectations are taken conditioning on training data D^n.

Proposition 1. *Suppose g is a twice continuously differentiable function, then we have*

$$
\begin{aligned}
\mathbb{E}[\widehat{\mathcal{L}}_g] - \mathcal{L}_g = &\mathbb{E}_{\mathbb{P}}\left[\nabla g\left(\widehat{\boldsymbol{\theta}}_{\ell}(\boldsymbol{X})\right)^T \left(\mathbb{E}_{\mathbb{P}}(\boldsymbol{L}(O;\widehat{\mathbb{P}}) \mid \boldsymbol{X}) + \widehat{\boldsymbol{\theta}}_{\ell}(\boldsymbol{X}) - \boldsymbol{\theta}_{\ell}(\boldsymbol{X})\right)\right] \\
&-\tfrac{1}{2}\mathbb{E}_{\mathbb{P}}\left[\left(\widehat{\boldsymbol{\theta}}_{\ell}(\boldsymbol{X}) - \boldsymbol{\theta}_{\ell}(\boldsymbol{X})\right)^T \nabla^2 g\left(\boldsymbol{\theta}_{\ell}^*(\boldsymbol{X})\right)\left(\widehat{\boldsymbol{\theta}}_{\ell}(\boldsymbol{X}) - \boldsymbol{\theta}_{\ell}(\boldsymbol{X})\right)\right],
\end{aligned}
$$

where $\boldsymbol{L}(O;\widehat{\mathbb{P}}) = \left(L_1(O;\widehat{\mathbb{P}}), \ldots, L_8(O;\widehat{\mathbb{P}})\right)^T$ and $\boldsymbol{\theta}_{\ell}^*(\boldsymbol{X})$ is a point that lies on the line segment between $\boldsymbol{\theta}_{\ell}(\boldsymbol{X})$ and $\widehat{\boldsymbol{\theta}}_{\ell}(\boldsymbol{X})$.

Proof. By definition of $\widehat{\mathcal{L}}_g$,

$$
\begin{aligned}
&\mathbb{E}[\widehat{\mathcal{L}}_g] - \mathcal{L}_g \\
=&\mathbb{E}_{\mathbb{P}}\left[g\left(\widehat{\boldsymbol{\theta}}_\ell(\boldsymbol{X})\right) + \sum_{j=1}^{8} \frac{\partial g\left(\widehat{\boldsymbol{\theta}}_\ell(\boldsymbol{X})\right)}{\partial \widehat{\theta}_{\ell,j}(\boldsymbol{X})} L_j(O;\widehat{\mathbb{P}}) - g\left(\boldsymbol{\theta}_\ell(\boldsymbol{X})\right)\right] \\
=&\mathbb{E}_{\mathbb{P}}\left[g\left(\widehat{\boldsymbol{\theta}}_\ell(\boldsymbol{X})\right) + \sum_{j=1}^{8} \frac{\partial g\left(\widehat{\boldsymbol{\theta}}_\ell(\boldsymbol{X})\right)}{\partial \widehat{\theta}_{\ell,j}(\boldsymbol{X})} \mathbb{E}_{\mathbb{P}}\left(L_j(O;\widehat{\mathbb{P}}) \mid \boldsymbol{X}\right) - g\left(\boldsymbol{\theta}_\ell(\boldsymbol{X})\right)\right] \\
=&\mathbb{E}_{\mathbb{P}}\left[\nabla g\left(\widehat{\boldsymbol{\theta}}_\ell(\boldsymbol{X})\right)^T \mathbb{E}_{\mathbb{P}}(\boldsymbol{L}(O;\widehat{\mathbb{P}}) \mid \boldsymbol{X}) - \left(g\left(\boldsymbol{\theta}_\ell(\boldsymbol{X})\right) - g(\widehat{\boldsymbol{\theta}}_\ell(\boldsymbol{X}))\right)\right] \\
=&\mathbb{E}_{\mathbb{P}}\left[\nabla g\left(\widehat{\boldsymbol{\theta}}_\ell(\boldsymbol{X})\right)^T \left(\mathbb{E}_{\mathbb{P}}[\boldsymbol{L}(O;\widehat{\mathbb{P}}) \mid \boldsymbol{X}] + \widehat{\boldsymbol{\theta}}_\ell(\boldsymbol{X}) - \boldsymbol{\theta}_\ell(\boldsymbol{X})\right)\right] \\
&- \frac{1}{2}\mathbb{E}_{\mathbb{P}}\left[\left(\widehat{\boldsymbol{\theta}}_\ell(\boldsymbol{X}) - \boldsymbol{\theta}_\ell(\boldsymbol{X})\right)^T \nabla^2 g\left(\boldsymbol{\theta}^*_\ell(\boldsymbol{X})\right)\left(\widehat{\boldsymbol{\theta}}_\ell(\boldsymbol{X}) - \boldsymbol{\theta}_\ell(\boldsymbol{X})\right)\right].
\end{aligned}
$$

The second equality follows from conditioning on $\boldsymbol{X}$ and the last equality follows from second-order Taylor expansion. □

For any $(y, a, z) \in \{0,1\}^3$, by conditioning on $(Z, \boldsymbol{X})$ we have

$$
\begin{aligned}
\mathbb{E}_{\mathbb{P}}\left(\psi_{ya.z}(O;\widehat{\mathbb{P}}) \mid \boldsymbol{X}\right) &= \mathbb{E}\left[\frac{\mathbb{1}(Z=z)}{\widehat{\lambda}_z(\boldsymbol{X})}\left(\mathbb{P}(Y=y, A=a \mid \boldsymbol{X}, Z=z) - \widehat{\pi}_{ya.z}(\boldsymbol{X})\right) \mid \boldsymbol{X}\right] \\
&= \frac{\lambda_z(\boldsymbol{X})}{\widehat{\lambda}_z(\boldsymbol{X})}\left\{\pi_{ya.z}(\boldsymbol{X}) - \widehat{\pi}_{ya.z}(\boldsymbol{X})\right\}.
\end{aligned}
$$

We want to bound each component of $\mathbb{E}_{\mathbb{P}}[\boldsymbol{L}(O;\widehat{\mathbb{P}}) \mid \boldsymbol{X}] + \widehat{\boldsymbol{\theta}}_\ell(\boldsymbol{X}) - \boldsymbol{\theta}_\ell(\boldsymbol{X})$. We only analyze the first component as an illustration. All other components can be similarly analyzed.

$$
\begin{aligned}
&\mathbb{E}_{\mathbb{P}}\left(L_1(O;\widehat{\mathbb{P}}) \mid \boldsymbol{X}\right) + \widehat{\theta}_{\ell,1}(\boldsymbol{X}) - \theta_{\ell,1}(\boldsymbol{X}) \\
=&\mathbb{E}_{\mathbb{P}}\left(\psi_{11.1}(O;\widehat{\mathbb{P}}) + \psi_{00.0}(O;\widehat{\mathbb{P}}) \mid \boldsymbol{X}\right) + \{\widehat{\pi}_{11.1}(\boldsymbol{X}) - \pi_{11.1}(\boldsymbol{X})\} + \{\widehat{\pi}_{00.0}(\boldsymbol{X}) - \pi_{00.0}(\boldsymbol{X})\} \\
=&\left\{1 - \frac{\lambda_1(\boldsymbol{X})}{\widehat{\lambda}_1(\boldsymbol{X})}\right\}\{\widehat{\pi}_{11.1}(\boldsymbol{X}) - \pi_{11.1}(\boldsymbol{X})\} + \left\{1 - \frac{\lambda_0(\boldsymbol{X})}{\widehat{\lambda}_0(\boldsymbol{X})}\right\}\{\widehat{\pi}_{00.0}(\boldsymbol{X}) - \pi_{00.0}(\boldsymbol{X})\}.
\end{aligned}
$$

Note that

$$
1 - \frac{\lambda_1(\boldsymbol{X})}{\widehat{\lambda}_1(\boldsymbol{X})} = \frac{\widehat{\lambda}_1(\boldsymbol{X}) - \lambda_1(\boldsymbol{X})}{\widehat{\lambda}_1(\boldsymbol{X})}, 1 - \frac{\lambda_0(\boldsymbol{X})}{\widehat{\lambda}_0(\boldsymbol{X})} = \frac{\lambda_1(\boldsymbol{X}) - \widehat{\lambda}_1(\boldsymbol{X})}{1 - \widehat{\lambda}_1(\boldsymbol{X})}
$$

By positivity assumption we have

$$
\begin{aligned}
&\left|\mathbb{E}_{\mathbb{P}}\left(L_1(O;\widehat{\mathbb{P}}) \mid \boldsymbol{X}\right) + \widehat{\theta}_{\ell,1}(\boldsymbol{X}) - \theta_{\ell,1}(\boldsymbol{X})\right| \\
\leq& \frac{1}{\epsilon}\left|\widehat{\lambda}_1(\boldsymbol{X}) - \lambda_1(\boldsymbol{X})\right| \cdot \{|\widehat{\pi}_{11.1}(\boldsymbol{X}) - \pi_{11.1}(\boldsymbol{X})| + |\widehat{\pi}_{00.0}(\boldsymbol{X}) - \pi_{00.0}(\boldsymbol{X})|\}
\end{aligned}
$$

Similar inequalities can be obtained for $2 \leq j \leq 8$. Hence, by Hölder's inequality,

$$\begin{aligned}
&\left|\mathbb{E}_{\mathbb{P}}\left(\nabla g\left(\widehat{\boldsymbol{\theta}}_{\ell}(\boldsymbol{X})\right)^T\left\{\mathbb{E}_{\mathbb{P}}(\boldsymbol{L}(O;\widehat{\mathbb{P}}) \mid \boldsymbol{X})+\widehat{\boldsymbol{\theta}}_{\ell}(\boldsymbol{X})-\boldsymbol{\theta}_{\ell}(\boldsymbol{X})\right\}\right)\right| \\
\leq &\mathbb{E}_{\mathbb{P}}\left[\left\|\nabla g\left(\widehat{\boldsymbol{\theta}}_{\ell}(\boldsymbol{X})\right)\right\|_{\infty}\left\|\mathbb{E}_{\mathbb{P}}(\boldsymbol{L}(O;\widehat{\mathbb{P}}) \mid \boldsymbol{X})+\widehat{\boldsymbol{\theta}}_{\ell}(\boldsymbol{X})-\boldsymbol{\theta}_{\ell}(\boldsymbol{X})\right\|_1\right] \\
\leq &\frac{C_1}{\epsilon}\mathbb{E}_{\mathbb{P}}\left[\left|\widehat{\lambda}_1(\boldsymbol{X})-\lambda_1(\boldsymbol{X})\right| \sum_{(y,a,z)\in\mathcal{R}}\left|\widehat{\pi}_{ya.z}(\boldsymbol{X})-\pi_{ya.z}(\boldsymbol{X})\right|\right],
\end{aligned}$$

where $\mathcal{R}$ is a multiset of elements in $\{0,1\}^3$ such that each $(y,a,z) \in \{0,1\}^3$ appears in $\mathcal{R}$ as many times as $\pi_{ya.z}(\boldsymbol{X})$ appears in $\boldsymbol{\theta}_{\ell}(\boldsymbol{X})$. By the triangle inequality and Cauchy-Schwarz's inequality

$$\begin{aligned}
&\left|\mathbb{E}_{\mathbb{P}}\left(\nabla g\left(\widehat{\boldsymbol{\theta}}_{\ell}(\boldsymbol{X})\right)^T\left\{\mathbb{E}_{\mathbb{P}}(\boldsymbol{L}(O;\widehat{\mathbb{P}}) \mid \boldsymbol{X})+\widehat{\boldsymbol{\theta}}_{\ell}(\boldsymbol{X})-\boldsymbol{\theta}_{\ell}(\boldsymbol{X})\right\}\right)\right| \\
\lesssim &C_1\left\|\widehat{\lambda}_1-\lambda_1\right\| \sum_{(y,a,z)\in\mathcal{R}}\left\|\widehat{\pi}_{ya.z}-\pi_{ya.z}\right\| \\
\lesssim &C_1\left\|\widehat{\lambda}_1-\lambda_1\right\|\left(\max_{y,a,z\in\{0,1\}}\left\|\widehat{\pi}_{ya.z}-\pi_{ya.z}\right\|\right)
\end{aligned}$$

It remains to bound the second derivative term in the asymptotic bias expression derived in Proposition 1. By properties of the operator norm we have

$$\begin{aligned}
&\left|\mathbb{E}_{\mathbb{P}}\left(\left(\widehat{\boldsymbol{\theta}}_{\ell}(\boldsymbol{X})-\boldsymbol{\theta}_{\ell}(\boldsymbol{X})\right)^T \nabla^2 g\left(\boldsymbol{\theta}_{\ell}^*(\boldsymbol{X})\right)\left(\widehat{\boldsymbol{\theta}}_{\ell}(\boldsymbol{X})-\boldsymbol{\theta}_{\ell}(\boldsymbol{X})\right)\right)\right| \\
\leq &\left\{\sup_{\boldsymbol{\theta}}\left\|\nabla^2 g(\boldsymbol{\theta})\right\|\right\}\mathbb{E}_{\mathbb{P}}\left(\left\|\widehat{\boldsymbol{\theta}}_{\ell}(\boldsymbol{X})-\boldsymbol{\theta}_{\ell}(\boldsymbol{X})\right\|_2^2\right) \\
\lesssim &C_2\max_{y,a,z\in\{0,1\}}\left\|\widehat{\pi}_{ya.z}-\pi_{ya.z}\right\|^2
\end{aligned}$$

The bound on conditional bias is established. For a general function f on the sample O, we have

$$\mathbb{P}_n[\widehat{f}]-\mathbb{E}[f]=(\mathbb{P}_n-\mathbb{E})(\widehat{f}-f)+(\mathbb{P}_n-\mathbb{E})(f)+\mathbb{E}(\widehat{f}-f) \tag{6}$$

We apply the decomposition of error (6) to

$$f(O)=\dot{\mathcal{L}}_g(O;\mathbb{P})+\mathcal{L}_g(\mathbb{P})=g\left(\boldsymbol{\theta}_{\ell}(\boldsymbol{X})\right)+\sum_{j=1}^{8}\frac{\partial g\left(\boldsymbol{\theta}_{\ell}(\boldsymbol{X})\right)}{\partial\theta_{\ell,j}(\boldsymbol{X})}L_j(O;\mathbb{P}),$$

Note that $\mathbb{P}_n[\widehat{f}]$ is exactly the robust estimator. By Lemma 2 in Kennedy et al. (2020), if $\|\widehat{f}-f\|_2=o_{\mathbb{P}}(1)$ we have

$$(\mathbb{P}_n-\mathbb{E})(\widehat{f}-f)=o_{\mathbb{P}}(n^{-1/2}).$$

Also note $\mathbb{E}(\widehat{f}-f)$ is equal to the conditional bias and under the convergence rate assumption we have $\mathbb{E}(\widehat{f}-f)=o_{\mathbb{P}}(n^{-1/2})$. Finally note that $f-\mathbb{E}f=\dot{\mathcal{L}}_g(O;\mathbb{P})$, the proof is completed.

S5 Proofs of Results in Section 6

S5.1 Proof of Theorem 5

First by Fubini's theorem and Jensen's inequality we have

$$\int_0^1\mathbb{E}\left[\min_{1\leq j\leq 8}\theta_{u,j}(t,\boldsymbol{X})\right]dt=\mathbb{E}\left[\int_0^1\min_{1\leq j\leq 8}\theta_{u,j}(t,\boldsymbol{X})dt\right]\leq\mathbb{E}\left[\min_{1\leq j\leq 8}\int_0^1\theta_{u,j}(t,\boldsymbol{X})dt\right].$$

The proof will be completed if we can show

$$\int_0^1 \theta_{u,j}(t, \boldsymbol{X})dt = \widetilde{\theta}_{u,j}(\boldsymbol{X}).$$

Since $\theta_{u,j}$'s are linear combinations of $\pi_{1a.z}$ and $\pi_{0a.z}$, we only need to show for any $a, z \in \{0,1\}$,

$$\int_0^1 \pi_{1a.z}(t, \boldsymbol{X})dt = \widetilde{\pi}_{1a.z}(\boldsymbol{X}),$$

$$\int_0^1 \pi_{0a.z}(t, \boldsymbol{X})dt = \widetilde{\pi}_{0a.z}(\boldsymbol{X}).$$

We condition on W, by property of conditional expectation,

$$\begin{aligned}
&\widetilde{\pi}_{1a.z}(\boldsymbol{X}) \\
=&\mathbb{P}(Y \leq W, A = a \mid Z = z, \boldsymbol{X}) \\
=&\int_0^1 \mathbb{P}(Y \leq t, A = a \mid Z = z, \boldsymbol{X}, W = t)p_w(t \mid Z = z, \boldsymbol{X})dt,
\end{aligned}$$

where p_w denotes the density of W. Since W is independent of the data generating process of $O = (\boldsymbol{X}, Z, A, Y)$, we have

$$\mathbb{P}(Y \leq t, A = a \mid Z = z, \boldsymbol{X}, W = t) = \mathbb{P}(Y \leq t, A = a \mid Z = z, \boldsymbol{X}),$$

$$p_w(t \mid Z = z, \boldsymbol{X}) = p_w(t) = \mathbb{1}(0 < t < 1).$$

Hence we conclude

$$\int_0^1 \mathbb{P}(Y \leq t, A = a \mid Z = z, \boldsymbol{X}, W = t)p_w(t \mid Z = z, \boldsymbol{X})dt = \int_0^1 \mathbb{P}(Y \leq t, A = a \mid Z = z, \boldsymbol{X})dt.$$

Note that by definition $\pi_{1a.z}(t, \boldsymbol{X}) = \mathbb{P}(Y \leq t, A = a \mid \boldsymbol{X}, Z = z)$, which implies

$$\int_0^1 \pi_{1a.z}(t, \boldsymbol{X})dt = \widetilde{\pi}_{1a.z}(\boldsymbol{X}).$$

We can similarly prove

$$\int_0^1 \pi_{0a.z}(t, \boldsymbol{X})dt = \widetilde{\pi}_{0a.z}(\boldsymbol{X}).$$

which completes the proof for the first inequality in Theorem 5. The second inequality can be proved by the same arguments.

S5.2 Basic Efficiency Result

Proposition 2. *Suppose we observe n iid copies of $T \sim P$, with $T \in [0,1]$. Letting $\mu = \mathbb{E}_P(T)$, $\mathrm{Var}_P(T) = \sigma^2$, construct m estimates of μ by sampling $m \times n$ independent* Unif(0, 1) *variates, $\{W_i^{(j)}\}_{i=1,\ldots,n}^{j=1,\ldots,m}$, and estimating $\widehat{\mu}_j = \mathbb{P}_n[T > W^{(j)}] = \frac{1}{n}\sum_{i=1}^n \mathbb{1}(T_i > W_i^{(j)})$. Then the estimator that averages these m estimates,*

$$\widehat{\mu} = \frac{1}{m}\sum_{j=1}^m \widehat{\mu}_j,$$

is unbiased and has variance $\frac{1}{n}\left(\sigma^2 + \frac{1}{m}\left\{\mu(1-\mu) - \sigma^2\right\}\right) \overset{m\to\infty}{\to} \frac{\sigma^2}{n}$.

Proof. Observe that $\mathbb{E}_P(\widehat{\mu}_j) = P[T > W] = \int_0^1 P[T > w]\, dw = \mathbb{E}_P(T) = \mu$, for $j = 1, \ldots, m$, so $\widehat{\mu}$ is unbiased. Further, $\mathrm{Var}_P(\widehat{\mu}_j) = \frac{1}{n}\mathrm{Var}_P(\mathbb{1}(T > W)) = \frac{1}{n}\mu(1-\mu)$, so by identical distribution of each $\widehat{\mu}_j$,

$$\begin{aligned}\mathrm{Var}_P(\widehat{\mu}) &= \frac{1}{m}\mathrm{Var}_P(\widehat{\mu}_1) + \left(1 - \frac{1}{m}\right)\mathrm{Cov}_P(\widehat{\mu}_1, \widehat{\mu}_2) \\ &= \frac{1}{nm}\mu(1-\mu) + \left(1 - \frac{1}{m}\right)\frac{1}{n}\mathrm{Cov}_P(\mathbb{1}(T > W^{(1)}), \mathbb{1}(T > W^{(2)}))\end{aligned}$$

using the fact that $\{W_i^{(j)}\} \perp\!\!\!\perp (T_1, \ldots, T_n)$. Next, see that

$$\mathrm{Cov}_P(\mathbb{1}(T > W^{(1)}), \mathbb{1}(T > W^{(2)})) = P[T > \max\{W^{(1)}, W^{(1)}\}] - \mu^2,$$

and finally, since $V = \max\{W^{(1)}, W^{(1)}\}$ has density $2v\mathbb{1}(v \in (0,1))$,

$$P[T > V] = \int_0^1 2vP[T > v]\, dv = \int_0^1 P[T > \sqrt{u}]\, du = \int_0^1 P[T^2 > u]\, du = \mathbb{E}_P(T^2) = \mu^2 + \sigma^2,$$

where we made the substitution $u = v^2$.

□

S6 Details of the Simulation Studies

S6.1 First Simulation Study

In our first simulation study, we consider the following data generating mechanism: two baseline covariates $\boldsymbol{X} = (X_1, X_2)$ are generated independently with $X_1 \sim \mathrm{Unif}(0,1)$ and $X_2 \sim \mathrm{Unif}(-1,1)$, and $Z \mid \boldsymbol{X} \sim \mathrm{Bernoulli}(\lambda_1(\boldsymbol{X}))$ where $\lambda_1(\boldsymbol{X}) = 0.35 + 0.6\{0.5X_1 + 0.2X_2\}$. The compliance classes are then determined from $\boldsymbol{X}$ as follows:

$$\begin{aligned} &A(z=0) = 1, A(z=1) = 0 \quad \text{when } X_1 \le 0.3, X_2 \ge 0 \\ &A(z=0) = 0, A(z=1) = 1 \quad \text{when } X_1 \ge 0.3, X_2 \ge -0.4 \\ &A(z=0) = 0, A(z=1) = 0 \quad \text{when } X_2 \le -0.4 \\ &A(z=0) = 1, A(z=1) = 1 \quad \text{otherwise}\end{aligned}$$

and further $A \equiv ZA(z=1) + (1-Z)A(z=0)$. Finally, $Y(a) \sim \mathrm{Bernoulli}(p_a)$ are generating according to the compliance classes:

$$p_a = \begin{cases} 0.75 - 0.40a, & \text{if } A(z=0)\{1 - A(z=1)\} = 1, \\ 0.25 + 0.60a, & \text{if } \{1 - A(z=0)\}A(z=1) = 1, \\ 0.20 + 0.10a, & \text{if } A(z=0)A(z=1) = 1, \\ 0.90 + 0.05a, & \text{if } \{1 - A(z=0)\}\{1 - A(z=1)\} = 1 \end{cases}$$

and $Y \equiv AY(a=1) + (1-A)Y(a=0)$. This scenario yields covariate adjusted Balke-Pearl bounds of $(\mathcal{L}(\mathbb{P}), \mathcal{U}(\mathbb{P})) = (-0.0784, 0.282)$.

In order to evaluate our methods, we simulated 500 datasets of size $n = 10000$ from the above data generating mechanism. We then computed naïve unadjusted Balke-Pearl bounds empirically, and constructed covariate-adjusted estimators $(\widehat{\mathcal{L}}, \widehat{\mathcal{L}}_{gt}, \widehat{\mathcal{U}}, \widehat{\mathcal{U}}_{gt})$ as proposed in Sections 4 and 5. Employing a single sample split, we constructed the proposed one-step estimators $(\widehat{\mathcal{L}}, \widehat{\mathcal{U}})$ using flexible ensembles for the nuisance functions. Specifically, we used ensembles

of generalized additive models (`gam`), regression trees (`rpart`), and the lasso (`glmnet`) using the `SuperLearner` package in `R` (Polley et al., 2019) to obtain $\{\widehat{\pi}_{ya.z}(\boldsymbol{X}) : y, a, z \in \{0, 1\}\}$, with each $\widehat{\pi}_{ya.z}$ being fit separately. A similar ensemble was used to fit $\widehat{\lambda}_1(\boldsymbol{X})$, but we additionally included a generalized linear model (`glm`). The LSE-based estimators $\widehat{\mathcal{L}}_{g_t}$ and $\widehat{\mathcal{U}}_{h_t}$ were constructed based on the same nuisance function estimators, using the *ad hoc* tuning parameter choice $t = 100\,(n/2)^{1/4}$.

In order to compare our methods to a more parametric approach, we also computed the estimators $(\widehat{\mathcal{L}}, \widehat{\mathcal{U}})$ exactly as above, but using logistic regression (with main effects for X_1 and X_2) for all the nuisance functions $\{\widehat{\pi}_{ya.z}(\boldsymbol{X}) : y, a, z \in \{0, 1\}\}$ and $\widehat{\lambda}_1(\boldsymbol{X})$.

The results of this first simulation study are compiled and displayed in Table S1. Namely, we show the empirical mean, bias, and standard error of each estimator across the 500 simulation replications. The proposed estimators with flexible covariate adjustment exhibit very little bias. On the other hand, the parametrically covariate-adjusted estimators are highly biased for the true lower and upper bound functionals, due to poor nuisance function estimation. Unsurprisingly, the unadjusted Balke-Pearl bounds are much wider than the adjusted bounds in this scenario, as the covariates help to drastically reduce the bound width in line with Corollary 1.

Estimator	Mean	Percent Bias (%)	Standard Error
Unadjusted lower bound	-0.160	104.1	0.010
Parametrically adjusted lower bound	-0.181	130.8	0.017
Flexibly adjusted lower bound ($\widehat{\mathcal{L}}$)	-0.0803	2.4	0.015
Approximation-based adjusted lower bound ($\widehat{\mathcal{L}}_{g_t}$)	-0.0804	2.5	0.015
Unadjusted upper bound	0.423	50.0	0.007
Parametrically adjusted upper bound	0.359	27.3	0.015
Flexibly adjusted upper bound ($\widehat{\mathcal{U}}$)	0.284	0.7	0.013
Approximation-based adjusted upper bound ($\widehat{\mathcal{U}}_{g_t}$)	0.284	0.7	0.013

Table S1: Results of first simulation study

S6.2 Second Simulation Study

The second simulation study considers a simplified setting: with $\boldsymbol{X} \equiv X \sim \text{Uniform}(0, 1)$, we generated $Z \sim \text{Bernoulli}(\lambda_1(X))$ where $\lambda_1(X) = \min\{\max\{X^2, 0.10\}, 0.90\}$. Next, we assumed perfect compliance, i.e., $A \equiv Z$, then generated $U \sim \text{Uniform}(0, 1)$, independent of (X, A), and $Y \mid X, A, U \sim \text{Bernoulli}\left(U\{A(1-X) + (1-A)X\}\right)$. This simplified setup, in which X is sufficient for controlling for $A - Y$ confounding, results in

$$\begin{aligned}\min_{j \neq d_\ell(X)} \left\{\theta_{\ell, d_\ell(X)}(X) - \theta_{\ell, j}(X)\right\} &= \min\{\mu_0(X), 1 - \mu_0(X), \mu_1(X), 1 - \mu_1(X)\} \\ &= \frac{1}{2}\min\{X, 1 - X\},\end{aligned}$$

where $\mu_a(X) = P[Y = 1 \mid X, A = a]$. In turn, this guarantees that Assumption 5 holds with $\alpha = 1$, since $\{X, 1 - X\} \sim \text{Uniform}\left(0, \frac{1}{2}\right)$:

$$P\left[\frac{1}{2}\min\{X, 1 - X\} \leq t\right] = \begin{cases} 0 & \text{if } t < 0 \\ 4t & \text{if } 0 \leq t \leq \frac{1}{4} \\ 1 & \text{if } t > \frac{1}{4} \end{cases}$$

which is at most $4t$ for all $t \geq 0$. Moreover, the conditional bounds satisfy $\gamma_\ell(X) = \gamma_u(X) = \frac{1}{2} - X$, and thus the marginal bounds are $\mathcal{L}(\mathbb{P}) = \mathcal{U}(\mathbb{P}) = 0$. In this scenario, the probabilities $\{\pi_{ya.z}(X) : y, a, z \in \{0,1\}\}$ are given by

$$\pi_{00.1}(X) = \pi_{10.1}(X) = \pi_{01.0}(X) = \pi_{11.0}(X) = 0,$$

$$\pi_{00.0}(X) = 1 - \frac{1}{2}X, \pi_{10.0}(X) = \frac{1}{2}X, \pi_{01.1}(X) = 1 - \frac{1}{2}(1-X), \pi_{11.1}(X) = \frac{1}{2}(1-X).$$

To "estimate" the nuisance functions, we define $\widehat{\lambda}_1(X) = \text{expit}\left(\text{logit}\left(\lambda_1(X)\right) + \epsilon_\lambda\right)$, $\widehat{\pi}_{ya.z} \equiv 0$ when $a \neq z$, and otherwise $\widehat{\pi}_{ya.z}(X) = \text{expit}\left(\text{logit}\left(\pi_{ya.z}(X)\right) + \epsilon_{ya.z}\right)$, where

$$\epsilon_\lambda, \epsilon_{00.0}, \epsilon_{10.0}, \epsilon_{01.1}, \epsilon_{11.1} \overset{\text{iid}}{\sim} \mathcal{N}(h\, n^{-r}, h^2\, n^{-2r}),$$

where we set $h = 2.25$, and vary the parameter $r \in (0, 0.5]$ in different scenarios. These choices guarantee that $\left\|\widehat{\lambda}_1 - \lambda_1\right\| = O(n^{-r})$ and $\|\widehat{\pi}_{ya.z} - \pi_{ya.z}\| = O(n^{-r})$ for $a = z$—as a consequence we can study the performance of the proposed estimators under different nuisance estimation convergence rates known to be $O(n^{-r})$.

Our simulation study proceeded as follows: for $n \in \{500, 1000, 5000\}$, we generated data and computed nuisance estimates $(\widehat{\lambda}_1, \{\widehat{\pi}_{ya.z} : y, a, z \in \{0,1\}\})$ as described above, varying $r \in \{0.10 + 0.05k : k \in \{0, \ldots, 8\}\}$. We then computed the direct lower bound estimator $\widehat{\mathcal{L}}$ as in Section 4, and the log-sum-exp smooth approximation-based estimator $\widehat{\mathcal{L}}_{g_t}$ as described in Section 5, taking $t = 2hn^r$. Finally, we computed a plug-in estimator $\mathbb{P}_n\left[\max_{1 \leq j \leq 8} \widehat{\theta}_{\ell,j}(X)\right]$, whose error is expected to be first order, i.e., on the order of the nuisance error $O(n^{-r})$. Each scenario was replicated $5,000$ times, and root-mean-square error (RMSE) of each estimator relative to the lower true bound $\mathcal{L}(\mathbb{P}) = 0$ was computed. Results are shown in Figure S1.

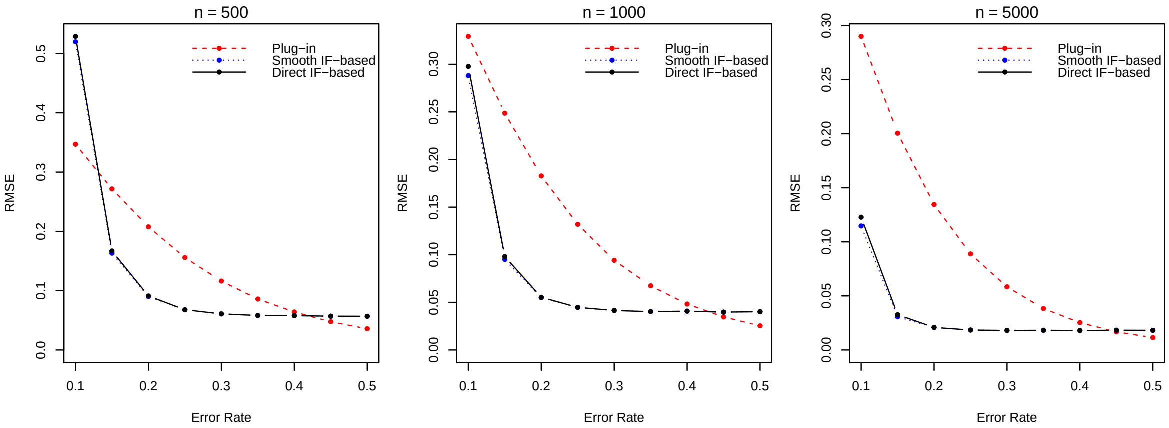

Figure S1: Root-mean-square error versus nuisance function error rate parameter r

According to the results in Figure S1, the proposed estimators $\widehat{\mathcal{L}}$ and $\widehat{\mathcal{L}}_{g_t}$ tend to outperform the plug-in estimator when nuisance functions are estimated slower than the parametric rate of $O(n^{-1/2})$, especially for larger sample sizes. Indeed, as predicted by our theoretical results, the robust estimators $\widehat{\mathcal{L}}$ and $\widehat{\mathcal{L}}_{g_t}$ nearly attained the optimal performance of the plug-in estimator (where nuisance error $\asymp n^{-1/2}$) when nuisance RMSE was only on the order $O(n^{-1/4})$, following which performance plateaued. As nuisance error on the order of $O(n^{-1/2})$ would be anticipated only in the unlikely scenario of a correctly specified parametric model,

the results support our recommendation to use the proposed estimators in practice. Note that in this simulation, the *ad hoc* choice for the tuning parameter t in $\widehat{\mathcal{L}}_{g_t}$ resulted in very little difference compared to $\widehat{\mathcal{L}}$, though other choices might be considered.

In this simulation study, we considered only the case $\alpha = 1$ from Assumption 5. In future work, we will consider simulation settings in which we can vary this margin parameter, and pursue theoretical development of optimal choices (e.g., in terms of minimizing MSE) for the tuning parameter t in $\widehat{\mathcal{L}}_{g_t}$.

S7 Code for Reproducing Motivating Illustration

```
library(rpart)

X1.p <- 0.7

beta.AT.0 <- function(x1, x2) {
  plogis(qlogis(0.20))
}
beta.AT.d <- function(x1, x2) {
  plogis(qlogis(0.30))
}
beta.NT.0 <- function(x1, x2) {
  plogis(qlogis(0.90))
}
beta.NT.d <- function(x1, x2) {
  plogis(qlogis(0.95))
}
beta.DE.0 <- function(x1, x2) {
  plogis(qlogis(0.65))
}
beta.DE.d <- function(x1, x2) {
  plogis(qlogis(0.70))
}
beta.CO.0 <- function(x1, x2) {
  plogis(qlogis(0.25))
}
beta.CO.d <- function(x1, x2) {
  plogis(qlogis(0.35))
}


## we work with an 8-vector pi = {pi_{ya.z} : y, a, z \in {0,1}}
## specifically, take the following order:
## pi = (pi_{00.0}, pi_{01.0}, pi_{10.0}, pi_{11.0},
##       pi_{00.1}, pi_{01.1}, pi_{10.1}, pi_{11.1})

## lower bound functions
p.l1 <- function(pi) { pi[8] + pi[1] - 1 }
```

```
p.l2 <- function(pi) { pi[4] + pi[5] - 1 }
p.l3 <- function(pi) { -pi[6] - pi[7] }
p.l4 <- function(pi) { -pi[2] - pi[3] }
p.l5 <- function(pi) { pi[4] - pi[8] - pi[7] - pi[2] - pi[3] }
p.l6 <- function(pi) { pi[8] - pi[4] - pi[3] - pi[6] - pi[7] }
p.l7 <- function(pi) { pi[5] - pi[6] - pi[7] - pi[2] - pi[1] }
p.l8 <- function(pi) { pi[1] - pi[2] - pi[3] - pi[6] - pi[5] }
gamma.l <- function(pi) { pmax(p.l1(pi), p.l2(pi), p.l3(pi), p.l4(pi),
                               p.l5(pi), p.l6(pi), p.l7(pi), p.l8(pi)) }

## upper bound functions
p.u1 <- function(pi) { 1 - pi[6] - pi[3] }
p.u2 <- function(pi) { 1 - pi[2] - pi[7] }
p.u3 <- function(pi) { pi[8] + pi[5] }
p.u4 <- function(pi) { pi[4] + pi[1] }
p.u5 <- function(pi) { -pi[2] + pi[6] + pi[5] + pi[4] + pi[1] }
p.u6 <- function(pi) { -pi[6] + pi[2] + pi[1] + pi[8] + pi[5] }
p.u7 <- function(pi) { -pi[7] + pi[8] + pi[5] + pi[4] + pi[3] }
p.u8 <- function(pi) { -pi[3] + pi[4] + pi[1] + pi[8] + pi[7] }
gamma.u <- function(pi) { pmin(p.u1(pi), p.u2(pi), p.u3(pi), p.u4(pi),
                               p.u5(pi), p.u6(pi), p.u7(pi), p.u8(pi)) }

set.seed(476)
N <- 1000000
X1 <- rbinom(N, 1, X1.p)
X2 <- runif(N, -1, 1)
cmp <- factor(ifelse(X1 == 0 & X2 >=-0.5 & X2 <= 0.5, "DE",
                     ifelse(X2 >= 0.99, "AT",
                            ifelse(X2 <= -0.99, "NT", "CO"))))

ATE.true <- (1 - X1.p)*0.5*0.05 + 0.005 * 0.05 + 0.005 * 0.10 +
  (1 - 0.01 - (1 - X1.p)*0.5)*0.10

## covariate-adjusted BP bounds
pi00.0 <- ((cmp == "NT") * (1 - beta.NT.0(X1, X2)) +
             (cmp == "CO") * (1 - beta.CO.0(X1, X2)))
pi01.0 <- ((cmp == "AT") * (1 - beta.AT.d(X1, X2)) +
             (cmp == "DE") * (1 - beta.DE.d(X1, X2)))
pi10.0 <- ((cmp == "NT") * beta.NT.0(X1, X2) +
             (cmp == "CO") * beta.CO.0(X1, X2))
pi11.0 <- ((cmp == "AT") * beta.AT.d(X1, X2) +
             (cmp == "DE") * beta.DE.d(X1, X2))
pi00.1 <- ((cmp == "NT") * (1 - beta.NT.0(X1, X2)) +
             (cmp == "DE") * (1 - beta.DE.0(X1, X2)))
pi01.1 <- ((cmp == "AT") * (1 - beta.AT.d(X1, X2)) +
             (cmp == "CO") * (1 - beta.CO.d(X1, X2)))
pi10.1 <- ((cmp == "NT") * beta.NT.0(X1, X2) +
             (cmp == "DE") * beta.DE.0(X1, X2))
```

```
pi11.1 <- ((cmp == "AT") * beta.AT.d(X1, X2) +
             (cmp == "CO") * beta.CO.d(X1, X2))

pi <- cbind(pi00.0, pi01.0, pi10.0, pi11.0,
            pi00.1, pi01.1, pi10.1, pi11.1)

cov.lower <- apply(pi, 1, gamma.l)
cov.upper <- apply(pi, 1, gamma.u)

pi.mean <- c(mean(pi00.0), mean(pi01.0), mean(pi10.0), mean(pi11.0),
             mean(pi00.1), mean(pi01.1), mean(pi10.1), mean(pi11.1))
mean.lower <- gamma.l(pi.mean)
mean.upper <- gamma.u(pi.mean)

c(mean(cov.lower), mean(cov.upper)) ## theoretical covariate-assisted BP bounds
c(mean.lower, mean.upper) ## theoretical covariate-agnostic BP bounds

par(mar = c(3,4,1,1))
plot(NULL, ylim = c(ATE.true - 0.24,ATE.true + 0.21), xlim = c(0.2,0.8),
     xlab = "", ylab = "Average Treatment Effect", xaxt = 'n')
axis(1, at = c(0.35, 0.65), labels = c("Covariate-Agnostic", "Covariate-Adjusted"))
abline(h = ATE.true, col = 'red', lty = 'dashed')
abline(h = 0)
arrows(x0=0.33, y0=mean.lower, x1=0.33, y1=mean.upper,
       code=3, angle=90, length=0.05, lwd=2,col = 'blue')
arrows(x0=0.63, y0=mean(cov.lower)-0.0007, x1=0.63, y1=mean(cov.upper)+0.0007,
       code=3, angle=90, length=0.05, lwd=2, col = 'blue')

## Simulated data
set.seed(956)
n <- 5000

X1 <- rbinom(n, 1, X1.p)
X2 <- runif(n, -1, 1)
cmp <- factor(ifelse(X1 == 0 & X2 >=-0.5 & X2 <= 0.5, "DE",
                     ifelse(X2 >= 0.99, "AT",
                            ifelse(X2 <= -0.99, "NT", "CO"))))

Z <- rbinom(n, 1, 0.5)
A <- ifelse(cmp == "NT", 0,
            ifelse(cmp == "AT", 1,
                   ifelse(cmp == "CO", Z, 1 - Z)))
Y.0 <- ifelse(cmp == "NT", rbinom(n, 1, beta.NT.0(X1, X2)),
              ifelse(cmp == "AT", rbinom(n, 1, beta.AT.0(X1, X2)),
                     ifelse(cmp == "CO", rbinom(n, 1, beta.CO.0(X1, X2)),
                            rbinom(n, 1, beta.DE.0(X1, X2)))))
Y.1 <- ifelse(cmp == "NT", rbinom(n, 1, beta.NT.d(X1, X2)),
              ifelse(cmp == "AT", rbinom(n, 1, beta.AT.d(X1, X2)),
```

```
                    ifelse(cmp == "CO", rbinom(n, 1, beta.CO.d(X1, X2)),
                           rbinom(n, 1, beta.DE.d(X1, X2)))))
Y <- A*Y.1 + (1 - A)*Y.0

dat <- cbind.data.frame(X1, X2, Z, A, Y)
dat$Y.A <- factor(dat$Y):factor(dat$A)

## Estimated covariate-agnostic BP-bounds
## compute estimated probabilities
pi.hat <- c(mean((1 - dat$A[dat$Z == 0]) * (1 - dat$Y[dat$Z == 0])),
            mean(dat$A[dat$Z == 0] * (1 - dat$Y[dat$Z == 0])),
            mean((1 - dat$A[dat$Z == 0]) * dat$Y[dat$Z == 0]),
            mean(dat$A[dat$Z == 0] * dat$Y[dat$Z == 0]),
            mean((1 - dat$A[dat$Z == 1]) * (1 - dat$Y[dat$Z == 1])),
            mean(dat$A[dat$Z == 1] * (1 - dat$Y[dat$Z == 1])),
            mean((1 - dat$A[dat$Z == 1]) * dat$Y[dat$Z == 1]),
            mean(dat$A[dat$Z == 1] * dat$Y[dat$Z == 1]))

## natural bounds
round(c(p.l1(pi.hat), p.u1(pi.hat)), 3)

## Balke & Pearl bounds
round(c(gamma.l(pi.hat), gamma.u(pi.hat)), 3)

## bootstrap CI's
B <- 1000
boot.res <- matrix(NA, nrow = B, ncol = 2)
for (b in 1:B) {
  dat.b <- dat[sample(1:n, n, replace = T),]
  pi.hat.b <- c(mean((1 - dat.b$A[dat.b$Z == 0]) * (1 - dat.b$Y[dat.b$Z == 0])),
                mean(dat.b$A[dat.b$Z == 0] * (1 - dat.b$Y[dat.b$Z == 0])),
                mean((1 - dat.b$A[dat.b$Z == 0]) * dat.b$Y[dat.b$Z == 0]),
                mean(dat.b$A[dat.b$Z == 0] * dat.b$Y[dat.b$Z == 0]),
                mean((1 - dat.b$A[dat.b$Z == 1]) * (1 - dat.b$Y[dat.b$Z == 1])),
                mean(dat.b$A[dat.b$Z == 1] * (1 - dat.b$Y[dat.b$Z == 1])),
                mean((1 - dat.b$A[dat.b$Z == 1]) * dat.b$Y[dat.b$Z == 1]),
                mean(dat.b$A[dat.b$Z == 1] * dat.b$Y[dat.b$Z == 1]))
  boot.res[b,] <- c(gamma.l(pi.hat.b), gamma.u(pi.hat.b))
}

arrows(x0=0.37, y0=quantile(boot.res[,1],0.025),
       x1=0.37, y1=quantile(boot.res[,2],0.975),
       code=3, angle=90, length=0.05, lwd=2,col = 'darkgreen')

## Estimated covariate-assisted BP-bounds
M <- 10 ## number of data splits
test.indices <- list()
remaining <- 1:n
```

```
for (m in 1:(M-1)) {
  test.indices[[m]] <- sample(remaining, floor(n/M), replace = F)
  remaining <- remaining[! (remaining %in% test.indices[[m]])]
}
test.indices[[M]] <- remaining

analysis <- function(dat.train, dat.test) {

  pi.ya.0.rf <- rpart(Y.A ~ X1 + X2, data = dat.train[dat.train$Z==0, ])
  dat.test <- cbind.data.frame(dat.test, predict(pi.ya.0.rf, type = 'prob',
                                              newdata = dat.test))
  ## compile pi.hat_{ya.0} estimates
  colnames(dat.test)[(ncol(dat.test) - 3):ncol(dat.test)] <-
    c("pi_00.0", "pi_01.0", "pi_10.0", "pi_11.0")

  pi.ya.1.rf <- rpart(Y.A ~ X1 + X2, data = dat.train[dat.train$Z==1, ])
  dat.test <- cbind.data.frame(dat.test, predict(pi.ya.1.rf, type = 'prob',
                                              newdata = dat.test))
  ## compile pi.hat_{ya.1} estimates
  colnames(dat.test)[(ncol(dat.test) - 3):ncol(dat.test)] <-
    c("pi_00.1", "pi_01.1", "pi_10.1", "pi_11.1")

  lambda.1 <- 0.5
  ## compile all the IF contributions
  dat.test$psi_00.0 <- dat.test$pi_00.0 +
    (1 - dat.test$Z) * ((1 - dat.test$Y) *
                         (1 - dat.test$A) - dat.test$pi_00.0) /
    (1 - lambda.1)
  dat.test$psi_01.0 <- dat.test$pi_01.0 +
    (1 - dat.test$Z) * ((1 - dat.test$Y) * dat.test$A - dat.test$pi_01.0) /
    (1 - lambda.1)
  dat.test$psi_10.0 <- dat.test$pi_10.0 +
    (1 - dat.test$Z) * (dat.test$Y * (1 - dat.test$A) - dat.test$pi_10.0) /
    (1 - lambda.1)
  dat.test$psi_11.0 <- dat.test$pi_11.0 +
    (1 - dat.test$Z) * (dat.test$Y * dat.test$A - dat.test$pi_11.0) /
    (1 - lambda.1)
  dat.test$psi_00.1 <- dat.test$pi_00.1 +
    dat.test$Z *
    ((1 - dat.test$Y) * (1 - dat.test$A) - dat.test$pi_00.1) / lambda.1
  dat.test$psi_01.1 <- dat.test$pi_01.1 +
    dat.test$Z * ((1 - dat.test$Y) * dat.test$A - dat.test$pi_01.1) / lambda.1
  dat.test$psi_10.1 <- dat.test$pi_10.1 +
    dat.test$Z * (dat.test$Y * (1 - dat.test$A) - dat.test$pi_10.1) / lambda.1
  dat.test$psi_11.1 <-dat.test$pi_11.1 +
    dat.test$Z * (dat.test$Y * dat.test$A - dat.test$pi_11.1) / lambda.1

  pi.hats <- dat.test[,(ncol(dat.test) - 15):(ncol(dat.test) - 8)]
```

```
  psi.hats <- dat.test[,(ncol(dat.test) - 7):ncol(dat.test)]
  p.l.hats <- cbind(p.l1(pi.hats)[,1], p.l2(pi.hats)[,1], p.l3(pi.hats)[,1],
                    p.l4(pi.hats)[,1], p.l5(pi.hats)[,1], p.l6(pi.hats)[,1],
                    p.l7(pi.hats)[,1], p.l8(pi.hats)[,1])
  p.u.hats <- cbind(p.u1(pi.hats)[,1], p.u2(pi.hats)[,1], p.u3(pi.hats)[,1],
                    p.u4(pi.hats)[,1], p.u5(pi.hats)[,1], p.u6(pi.hats)[,1],
                    p.u7(pi.hats)[,1], p.u8(pi.hats)[,1])
  L.hats <- cbind(p.l1(psi.hats)[,1], p.l2(psi.hats)[,1], p.l3(psi.hats)[,1],
                  p.l4(psi.hats)[,1], p.l5(psi.hats)[,1], p.l6(psi.hats)[,1],
                  p.l7(psi.hats)[,1], p.l8(psi.hats)[,1])
  U.hats <- cbind(p.u1(psi.hats)[,1], p.u2(psi.hats)[,1], p.u3(psi.hats)[,1],
                  p.u4(psi.hats)[,1], p.u5(psi.hats)[,1], p.u6(psi.hats)[,1],
                  p.u7(psi.hats)[,1], p.u8(psi.hats)[,1])

  argmax.p.l <- apply(p.l.hats, MARGIN = 1, FUN = which.max)

  argmin.p.u <- apply(p.u.hats, MARGIN = 1, FUN = which.min)

  IF.l <- sapply(1:nrow(dat.test), function(i) { L.hats[i, argmax.p.l[i]] },
                 simplify = 0)
  IF.u <- sapply(1:nrow(dat.test), function(i) { U.hats[i, argmin.p.u[i]] },
                 simplify = 0)

  ## IF-based Balke & Pearl bounds

  return(c(lower = mean(IF.l), upper = mean(IF.u),
           lower.var = var(IF.l), upper.var = var(IF.u)))

}

results.BP <- lapply(test.indices, function(inds) {
  analysis(dat.train = dat[-inds,], dat.test = dat[inds,])
})
BP.low <- mean(sapply(results.BP, function(x) {x[1]}, simplify = 0))
BP.upp <- mean(sapply(results.BP, function(x) {x[2]}, simplify = 0))
BP.low.var <- mean(sapply(results.BP, function(x) {x[3]}, simplify = 0))
BP.upp.var <- mean(sapply(results.BP, function(x) {x[4]}, simplify = 0))

arrows(x0=0.67, y0=BP.low + qnorm(0.025) * sqrt(BP.low.var / n),
       x1=0.67, y1=BP.upp + qnorm(0.975) * sqrt(BP.upp.var / n),
       code=3, angle=90, length=0.05, lwd=2, col = 'darkgreen')
```

References

Aldous, D. (2005), “Spin Glasses: A Challenge for Mathematicians,” .

Audibert, J.-Y. and Tsybakov, A. B. (2007), "Fast learning rates for plug-in classifiers," *The Annals of Statistics*, 35, 608–633.

Balke, A. and Pearl, J. (1993), "Nonparametric bounds on causal effects from partial compliance data," Tech. Rep. Technical Report R–199–J, University of California, Los Angeles.

— (1997), "Bounds on treatment effects from studies with imperfect compliance," *Journal of the American Statistical Association*, 92, 1171–1176.

Bibaut, A. F. and van der Laan, M. J. (2017), "Data-adaptive smoothing for optimal-rate estimation of possibly non-regular parameters," *arXiv preprint arXiv:1706.07408*.

Bickel, P. J., Klaassen, C. A., Bickel, P. J., Ritov, Y., Klaassen, J., Wellner, J. A., and Ritov, Y. (1993), *Efficient and adaptive estimation for semiparametric models*, vol. 4, Springer.

Branson, Z., Kennedy, E. H., Balakrishnan, S., and Wasserman, L. (2023), "Causal effect estimation after propensity score trimming with continuous treatments," *arXiv preprint arXiv:2309.00706*.

Calafiore, G. C., Gaubert, S., and Possieri, C. (2020), "A universal approximation result for difference of log-sum-exp neural networks," *IEEE transactions on neural networks and learning systems*, 31, 5603–5612.

Chernozhukov, V., Chetverikov, D., Demirer, M., Duflo, E., Hansen, C., Newey, W., and Robins, J. M. (2018), "Double/debiased machine learning for treatment and structural parameters," *The Econometrics Journal*, 21, C1–C68.

Chernozhukov, V., Chetverikov, D., and Kato, K. (2012), "Central limit theorems and multiplier bootstrap when p is much larger than n," Tech. rep., cemmap working paper.

Fan, Y., Guerre, E., and Zhu, D. (2017), "Partial identification of functionals of the joint distribution of "potential outcomes"," *Journal of econometrics*, 197, 42–59.

Kennedy, E. H. (2016), "Semiparametric theory and empirical processes in causal inference," in *Statistical causal inferences and their applications in public health research*, Springer, pp. 141–167.

Kennedy, E. H., Balakrishnan, S., and G'Sell, M. (2020), "Sharp instruments for classifying compliers and generalizing causal effects," *The Annals of Statistics*, 48, 2008–2030.

Luedtke, A. R. and van der Laan, M. J. (2016), "Statistical inference for the mean outcome under a possibly non-unique optimal treatment strategy," *Annals of Statistics*, 44, 713.

Makarov, G. (1982), "Estimates for the distribution function of a sum of two random variables when the marginal distributions are fixed," *Theory of Probability & its Applications*, 26, 803–806.

Manski, C. F. (1990), "Nonparametric bounds on treatment effects," *The American Economic Review*, 80, 319–323.

Murphy, S. A. (2003), "Optimal dynamic treatment regimes," *Journal of the Royal Statistical Society: Series B (Statistical Methodology)*, 65, 331–355.

Nesterov, Y. (2005), "Smooth minimization of non-smooth functions," *Mathematical programming*, 103, 127–152.

Polley, E., LeDell, E., Kennedy, C., Lendle, S., and van der Laan, M. (2019), "Package 'SuperLearner'," *CRAN*.

Qian, M. and Murphy, S. A. (2011), "Performance guarantees for individualized treatment rules," *Annals of Statistics*, 39, 1180.

Robins, J. (1986), "A new approach to causal inference in mortality studies with a sustained exposure period—application to control of the healthy worker survivor effect," *Mathematical modelling*, 7, 1393–1512.

Robins, J., Li, L., Tchetgen, E., van der Vaart, A., et al. (2008), "Higher order influence functions and minimax estimation of nonlinear functionals," in *Probability and statistics: essays in honor of David A. Freedman*, Institute of Mathematical Statistics, pp. 335–421.

Robins, J. M. (1989), "The analysis of randomized and non-randomized AIDS treatment trials using a new approach to causal inference in longitudinal studies," *Health Service Research Methodology: a Focus on AIDS*, 113–159.

Sachs, M. C., Jonzon, G., Sjölander, A., and Gabriel, E. E. (2022), "A general method for deriving tight symbolic bounds on causal effects," *Journal of Computational and Graphical Statistics*, 1–23.

Scharfstein, D. O., Rotnitzky, A., and Robins, J. M. (1999), "Adjusting for nonignorable drop-out using semiparametric nonresponse models," *Journal of the American Statistical Association*, 94, 1096–1120.

Semenova, V. (2023), "Adaptive Estimation of Intersection Bounds: a Classification Approach," *arXiv preprint arXiv:2303.00982*.

Tsiatis, A. A. (2006), *Semiparametric theory and missing data*, Springer.

van der Vaart, A. W. (2000), *Asymptotic statistics*, vol. 3, Cambridge university press.

Vershynin, R. (2018), *High-dimensional probability: An introduction with applications in data science*, vol. 47, Cambridge university press.

Wainwright, M. J. (2019), *High-dimensional statistics: A non-asymptotic viewpoint*, vol. 48, Cambridge University Press.

Zheng, W. and van der Laan, M. J. (2010), "Asymptotic theory for cross-validated targeted maximum likelihood estimation," Tech. rep., U.C. Berkeley.
